# Index or illusion: The case of frailty indices in the Health and Retirement Study

**DOI:** 10.1371/journal.pone.0197859

**Published:** 2018-07-18

**Authors:** Yi-Sheng Chao, Hsing-Chien Wu, Chao-Jung Wu, Wei-Chih Chen

**Affiliations:** 1 Centre de recherche du centre hospitalier de l’Université de Montréal (CRCHUM), Université de Montréal, Montréal, Québec, Canada; 2 Taipei Hospital, Ministry of Health and Welfare, New Taipei city, Taiwan; 3 Département d'informatique, Université du Québec à Montréal, Montréal, Québec, Canada; 4 Department of Chest Medicine, Taipei Veterans General Hospital, Taipei, Taiwan; 5 Faculty of Medicine, School of Medicine, National Yang-Ming University, Taipei, Taiwan; Berner Fachhochschule, SWITZERLAND

## Abstract

**Introduction:**

Frailty is a geriatric syndrome that has been defined differently with various indices. Without a uniform definition, it remains unclear how to interpret and compare different frailty indices (FIs). With the advances in index mining, we find it necessary to review the implicit assumptions about the creation of FIs. We are concerned the processing of frailty data may introduce measurement error and bias. We aim to review the assumptions, interpretability and predictive power of FIs regarding mortality.

**Methods:**

Three FIs, the Functional Domains Model proposed by Strawbridge et al. (1998), the Burden Model by Rockwood et al. (2007) and the Biologic Syndrome Model by Fried et al. (2004), were directly compared using the data from the Health and Retirement Study (HRS), a longitudinal study since 1996 mainly following up Americans aged 50 years and over. The FIs were reproduced according to Cigolle et al. (2009) and interpreted with their input variables through forward-stepwise regression. Biases were the residuals of the FIs that could not be explained by own input variables. Any four of the input variables were used to create alternative indices. Discrete-time survival analysis was conducted to compare the predictive power of FIs, input variables and alternative indices on mortality.

**Results:**

We found frailty a syndrome not unique to the elderly. The FIs were produced with different degrees of bias. The FIs could not be fully interpreted with the theory-based input variables. The bias induced by the Biological Syndrome Model better predicted mortality than frailty status. A complicated FI, the Burden Model, could be simplified. The input variables better predicted mortality than the FIs. The continuous FIs predicted mortality better than the frailty statuses. At least 6865 alternative indices better predicted mortality than the FIs.

**Conclusion:**

FIs have been used as outcome in clinical trials and need to be reviewed for adequacy based on our findings. The three FIs are not closely linked to the theories because of bias introduced by data manipulation and excessive numbers of input variables. We are developing new algorithms to develop and validate innovative indices.

## Introduction

Frailty is defined as a geriatric syndrome and has been described with different measurement tools and theories[1–4]. Frailty has been defined with at least three different indices. Strawbridge et al. (1998) described frailty in the Functional Domain Model as “a grouping of problems and losses of capability which make the individual more vulnerable to environmental challenge”[1]. The four frailty domains are physical, nutritive, and cognitive functioning, and sensory problems[1]. The input variables to create a frailty index include dizziness, difficulty in lifting weights, weight loss and being underweight, cognitive impairment, poor hearing, and poor eyesight. Rockwood et al. (2007) developed measures of deficit accumulation to represent frailty in the Burden Model[3, 5]. The eligible deficits are symptoms or signs or conditions related to aging and there are 70 items selected[3]. Fried et al. (2004) interpreted frailty as “a biologic syndrome of decreased reserve and resistance to stressors, resulting from cumulative declines across multiple physiologic systems” in the Biological Syndrome Model[6]. The frailty criteria include weight loss, exhaustion, low energy expenditure, slowness, and weakness[6]. The input variables selected to represent the criteria are weight loss, underweight, feeling everything an effort, inability to get going, kilocalories of physical expenditure, time to walk 15 feet, and grip strengths[6]. Despite the differences in definitions and measurement, the concept of frailty has been proven useful to predict adverse health outcomes, such as mortality[7], falls[8], hospitalization[9], and surgical outcomes[10]. The biological mechanism of frailty, especially the concurrence of sarcopenia, has also been reviewed[11]. The concept of frailty has also been extended and serves as an outcome itself in many trials[12–14]. However, there are several concerns regarding how frailty indices are generated.

The concept of frailty has been criticized for its vagueness[15]. However, we speculate that the created frailty indices may not be connected to the fundament theories because of poor practices in index mining and data distortion. Our concerns are related to implicit assumption imposed by the criteria to generate frailty indices, equal weighting schemes for each candidate domain of frailty, and data processing that may be prone to the introduction of bias according to the principles of index mining (see S1 Appendix for a list of problems identified based on literature review and data analysis)[16].

Specifically, the first issue is a lack of criteria to exclude highly correlated or duplicate measures. This raises the concern about over-emphasizing particular functional domains or attributes[16]. For example, feeling happy and feeling depressed are both contributing to the Burden Model[3]. For the same index, “other medical history” can also be counted as a deficit[17]. It is very likely to lead patients to report conditions similar to other deficits already reported, but named differently. For example, patients may report metabolic syndrome as an additional deficit while its diagnostic criteria, obesity, hypertension and diabetes, are already counted as deficits.

Second, a similar issue is how to assign weights to the candidate attributes of frailty. The conventional wisdom is to assign equal weights to the eligibility criteria[2–4, 16]. However, applying equal weighting to the input variables of frailty indices is restricting the predictive power of the newly generated indices[16]. Even the creators of the Burden Model discover that the index consisting of measures assigned with equal weights does not predict mortality risk better than the index consisting of variables assigned with unequal weights[18].

Third, ordinal variables are often scaled within the range of zero and one by division[3]. They are directly taken as interval variables without further validating whether the intervals between all categories can be treated as equal in the Burden Model[3, 19]. In fact, conversion between ordinal variables and continuous measures requires extensive research that may be lacking in the generation of frailty index. For example, rescaling Likert scales to continuous visual analogue scale requires repetitive measurements of both scales in the same population to understand conversion feasibility and relationships between two scales[20].

**Fig 1:**
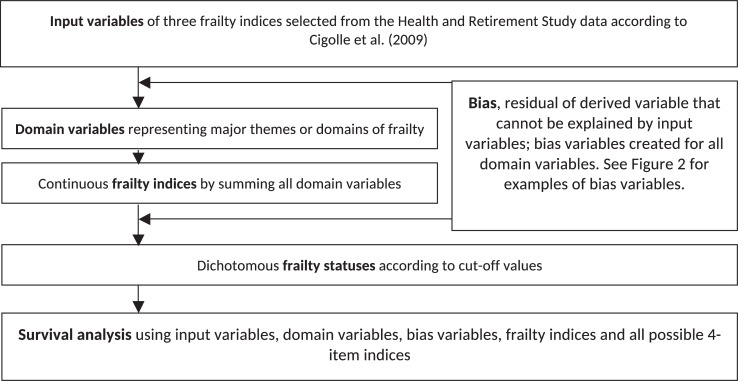
The flowchart of replicating and interpreting three frailty indices.

**Fig 2:**
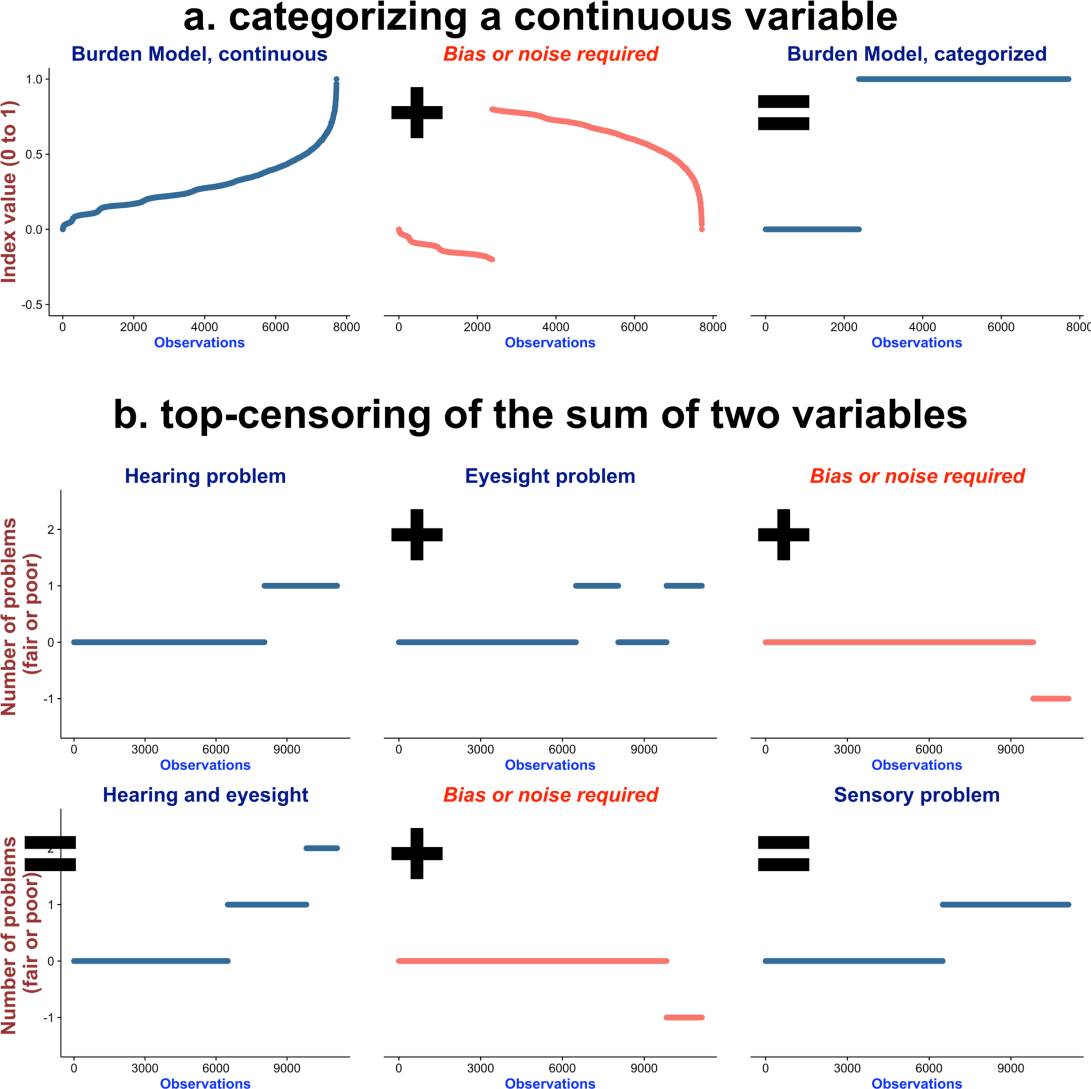
Examples of data manipulation that can introduce bias to the derived variables. Note. (a) this shows the relationship between an input continuous variable and the derived dichotomized variable. The observations were sorted by increasing order based on the values of the continuous variable, the frailty index in the Burden Model in this case. The horizontal axis was the number of the observations. The vertical axis was the value of the continuous index. The bias variable was the essential information that was not related to original input variable. For example, one observation had a value of 0.1 and the other had 0.9, while the cut-off threshold was 0.2. The values of the bias variable required for the two observations were -0.1 and 0.1 respectively to derive their statuses, non-frail (coded as zero) and frail (coded as one). (b) the sensory problem was defined by having problem of hearing or eyesight. The observations were sorted by having problem of hearing and eyesight. Those having problem of hearing and eyesight needed to be subtracted by one for having two problems. The negative values assigned to those having two problems were the bias variable generated.

Fourth, continuous variables are often categorized to discrete variables that are later summed to form a frailty index. However, categorizing continuous variables can introduce noise or even bias to original information[21]. While this practice is important to the generation of frailty indices[2], frailty indices may consist of information unrelated to original data (see Fig 1 for the steps to reproduce frailty indices and Fig 2 for illustration of bias introduced purely due to data manipulation).

Lastly, survey design may not have been properly considered in the Biological Syndrome Model. Unweighted survey data may be used to calculate percentiles that are later applied to mimic weighted percentiles and derive weighted statistics. We think this is the reason why there are 30% of the weighted samples with the slowest 20% time to walk eight feet and 21% of the weighted population with the weakest 20% grip strength[2].

These problems and the various frailty definitions prevent the readers from understanding the exact meaning of frailty. These issues need to be reviewed as soon as possible since the idea of frailty has been growing in popularity and used as an outcome in clinical trials. If the measurement of frailty is not valid, patients may receive unnecessary or even harmful treatment. To understand what frailty means in relationship to its input information, this study aims to replicate and reanalyze the three frailty indices with the publicly available HRS data[2]. Furthermore, in order to investigate whether these problems are also prevalent to other indices, we use Body Mass Index (BMI) as comparison. The objectives are to 1) test the reproducibility of frailty indices in Cigolle et al. (2009), 2) interpret continuous frailty indices, dichotomous frailty status and BMI with input variables in the HRS data, 3) understand the predictive power of existing frailty indices and BMI regarding mortality since its significant association with mortality has been the rationale to adopt the concept of frailty[6, 7], 4) search for alternative indices that better predict mortality, and 5) develop a preliminary guide to report the process of index mining based on the lessons we learn from the frailty indices.

## Methods

This secondary data analysis was approved by the ethics review committee at the Centre Hospitalier de l’Université de Montréal. The publicly available HRS data were full anonymized and de-identified before we accessed them. This study first reproduced the three frailty indices that were directly compared in Cigolle et al. (2009)[2] and interpreted frailty indices with input variables that were used to create them. Since the information published was not exhaustive and the author could not be reached, we designed a replication process to ensure the best replication of frailty indices with the HRS data. The procedures included 1) selecting and editing input variables for frailty indices according to the published information, while missing values were taken as separate categories[22], 2) creating temporary indices to see whether that sample sizes and frailty prevalence rates were similar to those published, 3) imputing missing values in input variables if the sample sizes and prevalence rates of the temporary indices were similar to those published, 4) recreating new indices with imputed data and comparing with temporary ones to assure the quality of data imputation, 5) assessing the differences in prevalence rates of new indices and the published ones, 6) creating finalized indices that were with acceptable differences in sample sizes and prevalence rates, 7) interpreting frailty indices with input, domain and bias variables, 8) evaluating the predictive power of indices, and input and bias variables regarding mortality, and 9) creating 4-item equal-weight alternative indices to understand the possible ranges of model fit and statistical significance in mortality prediction.

The HRS began in 1996 and followed up adults aged 50 years and over every two years in the United States[23]. The HRS data were first released by waves. This study used the longitudinal data set from the HRS with contribution from RAND Corporation, version P[23, 24]. This longitudinal data set merged all available waves since 1996 and included most of the variables from original wave-specific data. Some variables that existed only in 2004 wave were reintroduced to the longitudinal file to reproduce the analysis in Cigolle et al. (2009) (see S2 Appendix for the list of variables)[2]. Further details of the HRS study design could be found elsewhere[23].

### Variable selection to create temporary frailty indices

There were three frailty indices in Cigolle et al. (2009)[2] to replicate: the Functional Domain model proposed by Strawbridge et al. (1998)[1], the Burden model by Rockwood et al. (2007) [3], and the Biological Syndrome model by Fried et al. (2004)[6]. The variables used to create the indices were described in detail except for the Burden Model. There was no information on the exact variable names and missing data imputation for the three frailty indices[2]. We searched the HRS codebooks and selected the variables that resembled those published (see S2 Appendix for the identified variable names and their definitions). The nine and ten input variables for the Functional Domain and the Biological Syndrome models respectively could be retrieved according to Cigolle et al. (2009). These variables were then processed to create four and five domains, each of which ranged from zero to one. These two indices were the sum of four and five domain variables respectively. To calculate the domain variables, it usually required two or more input variables. For example, one of the domain variables for the Biological Syndrome model was “slowness” and measured by “time to walk eight feet, converted to time to walk 15 feet categorized by height and sex”[6]. Therefore, a slowness variable was created to represent this domain according to information from three variables: time to walk eight feet, sex and height. There were four and five domain variables in respective models, but one (weight loss) was shared by both. This led to a total of eight derived domain variables for both models.

The 38 measures in the HRS[2] to approximate the 70 items in the original Burden Model study[3] by Cigolle et al. (2009) could not be fully identified. Instead, we could only retrieve 24 HRS variables to represent 30 items included in the Burden Model[17]. According to the originally proposed method, the variables were scaled within the ranges of zero and one[19]. For example, if the input variables were dichotomous, the values were transformed to zero and one. If they were five categories, the derived values were zero, 0.25, 0.5, 0.75 and one[19]. The sum of all items scaled between zero and one were then summed and scaled between zero and one by dividing by 24, the actual number of HRS variables[19]. For the Burden Model, the cognition domain was calculated with two input variables, performance-based scores and proxy evaluation of interviewee cognition. One domain variable representing cognition was created for the Burden Model.

The frailty indices of these three models ranged from zero to four, from zero to one, and from zero to five respectively in Table 1. The cut-off values to determine the status of frailty were greater than or equal to two, 0.2, and three respectively[2]. The HRS combined respondent and nursing home resident weights were applied in order to adjust for complex survey design and generate weighted frailty prevalence rates. If sample sizes and weighted prevalence rates were the same or similar to those published, the indices were successfully replicated based on published information. However, weighting was only applied for the comparison of prevalence rates between this study and Cigolle et al. (2009). Other statistics remained unweighted.

**Table 1:** Characteristics of frailty indices and body mass index and the issues identified after the replication and the approximation of frailty indices with input variables.

Basic characteristics of frailty indices	1. Functional Domains Model	2. Burden Model	3. Biologic Syndrome Model	Body Mass Index
**Numbers of domains or items**	4	24 (representing 30 of original 70 items; 38 used in Cigolle et al. (2009))	5	1
**Numbers of variables required**	9	25	10	2
**Numbers of sources of bias due to data manipulation**	4	24	5	1
**Sample sizes in Cigolle et al. (2009)**	11,113	7,719	1,657	
**Inclusion criteria in Cigolle et al. (2009)**	Adults aged 65 and older	Adults aged 70 and older	Adults aged 65 and older who completed the performance measures and did not have stroke, depression, or moderate to severe cognitive impairment	
**Weighted frailty prevalence in Cigolle et al. (2009) (%)**	29%	32%	11%	
**Samples sizes retrieved in this study (n)**	11113	7713	1642	19750
**Weighted frailty prevalence in this study (%)**	26.8%	42.3% or 27.6% (divided by 24 variables or 30 items represented)	11.2%	
**Complete cases (n)**	9314	6635	1429	19750
**Proportions of complete cases**	0.8381	0.8602	0.8703	1.00
**Weighted frailty prevalence based on imputed data**	26.8%	43.5% or 29.3% (divided by 24 variables or 30 items represented)	10.6%	
**Age eligibility**	65 years and over	70 years and over	65 years and over	
**Unweighted prevalence of those meeting age criteria**	0.275	0.446	0.167	
**Unweighted prevalence of those younger than age criteria (minimal age: 24 years)**	0.138	0.239	0.1	
**Proportions of variances not explained by input variables**	0.1475	0.00015	0.2237	0.0058
**Numbers of variables used to approximate continuous frailty indices**	70	72	66	2
**Numbers of sources of bias due to data manipulation**	4	1	5	1
**Maximal proportions of variances of continuous frailty indices explained by own input variables (R squared)**	0.753	0.978	0.591	0.994
**Numbers of own input variables required for maximal R squared**	17	48	14	3
**Maximal proportions of variances of continuous frailty indices explained by all input variables (R squared)**	0.852	0.99985	0.776	0.994
**Numbers of all input variables required for maximal R squared**	63	58	42	3
**Maximal proportions of variances of continuous frailty indices explained by bias alone (R squared)**	0.265	<0.001	0.719	0.006
**Numbers of bias sources for maximal R squared**	4	1	5	1
**Numbers of variables used to approximate dichotomous frailty status**	49	49	45	2
**Proportions of variances explained by four leading input variables**	0.559	0.667	0.541	
**Four leading input variables to interpret frailty indices**	1) Physical functioning: difficulty lifting 10 pounds; 2) History relevant to cognitive impairment or loss; 3) Sensory problems: fair or poor hearing despite use of hearing aids; 4) Physical functioning: difficulty lifting 10 pounds	1) History of stroke; 2) Depression (clinical impression); 3) Impaired mobility; 4) Urinary incontinence	1) Felt that everything I did was an effort in last week.; 2) Could not get going in last week.; 3) Low energy expenditure: Frequency of three intensities of activity, lowest quintile (stratified according to sex); 4) Weakness: Grip strength: Weakest 20% (stratified according to sex and BMI)	
**Numbers of variables to achieve maximal AIC**	27	54	25	3
**AUC of best fit models (95% CIs) with input variables**	0.973 (0.971 to 0.976)	0.967 (0.963 to 0.97)	0.965 (0.955 to 0.975)	1 (1 to 1)
**AUC of best fit models (95% CIs) with bias variables**	0.755 (0.743 to 0.767)	0.44 (0.426 to 0.455)	0.968 (0.959 to 0.976)	0.522 (0.513 to 0.531)
**Complex frailty index could be simplified**		+		
**Number of input variables to explain 90% of the variance of continuous indices**		11		
**Number of input variables to explain 95% of the variance of continuous indices**		14		
**Number of input variables to explain 99% of the variance of continuous indices**		20		

Note: AIC = Akaike Information Criterion; AUC = area under curve; HRS = Health and Retirement Study. Body Mass Index (BMI) is dichotomized into two categories, overweight/obesity (BMI>25) and underweight/normal.

### Included participants and missing value imputation

We applied the same inclusion criteria, but there are slight differences in the numbers of eligible participants for three models (Table 1 and the details in S1 Appendix). Three temporary frailty indices were generated by summing numbers of deficits that were represented by eligible categories among included participants. With the sample sizes and weighted prevalence rates of three temporary frailty indices similar or the same to those published, the samples for frailty index replication were finalized.

We identified that all of the input variables of the three indices had missing values (see S2 Appendix for the numbers and proportions of participants with missing values for each frailty index and each variable). We assumed that three frailty indices were generated without any imputation in Cigolle et al. (2009). We also assumed that the missing values were grouped together as separate categories for all input variables in Cigolle et al. (2009). This was similar to one type of data processing used by some epidemiological studies[25].

Less than 87% of the eligible participants in each frailty model had complete information on the input variables (Table 1). After confirming that the differences in sample sizes and prevalence rates were acceptable, the missing values among the included participants were imputed by chained equations[26]. Imputed data were used to recalculate the finalized indices.

### Bias introduced in the process of creating domain variable

Biases were defined as the information of domain variables that could not be accounted for by the theory-based input variables that were used to create them. They represented the information unrelated to input variables and introduced only due to data processing and manipulation. Two methods about how bias or noise was introduced were visualized in Fig 2. For example, sensory problem was defined by having fair or poor hearing or eyesight. The values of these two items, zero or one, were summed and then right-censored to have maximal values as one. This meant that sensory problem, right-censored sum of two variables, was not linearly related to both items. If regressed with both items, there would be some information that could not be explained by either input variable. Since this type of explicit censoring induced the loss of information[27], we considered the creation of domain variables were associated with introduction of bias to frailty indices. The bias variables were unrelated to input variables and merely products of data manipulation, variable categorization or top censoring, but essential to the generation of frailty indices.

While domain variables were created, bias variables were also determined as the differences between domain variables and the values fitted with input variables, the residuals of derived domain variables that could not be explained by input variables. Because there were four, one and five domain variables created and one was common to two of three indices, nine bias variables were produced (see Table 1 for the numbers of domain variables).

### Interpretation of frailty indices and frailty statuses

Frailty in continuous scales and dichotomous states were approximated or interpreted with original input variables. For continuous frailty indices, we used forward-stepwise linear regression[28, 29] to select the set of input variables that best explained frailty indices. The model fit was assessed with Akaike Information Criterion (AIC)[29]. In addition to the input variables of all frailty indices, age, sex and race/ethnicity were also added as independent variables to interpret frailty indices. If frailty indices could be fully explained by input variables, the R squared should be one. On the contrary, if none of the variances of generated indices could be explained by input variables, the R squared would be zero. If R squared fell between zero and one, part of the frailty index variance could only be explained by the bias introduced during data processing and manipulation.

The frailty state or status (dichotomous, yes or no) proposed by the three models was interpreted with forward-stepwise logistic regression[28, 30]. The model fit was assessed with adjusted R squared[30]. The receiver operating characteristic (ROC) curve and the area under curve (AUC) with 95% confidence intervals (CIs)[30] of the best performing models were shown to understand the relationships between input variables and frailty statuses. All input or domain variables that appeared in three frailty indices could be used in forward selection (see Table 1 for the numbers of eligible variables; see S2 Appendix for the characteristics of input variables).

### Discrete-time survival analysis

Survival analysis was conducted to understand the predictive power of frailty indices, frailty statuses, bias variables, domain variables and input variables. The outcome was mortality among HRS participants interviewed in 2004. To do so, the last interview dates and death dates occurring after interview dates in 2004 were retrieved and recoded to a time-to-event variable. Death events were identified and labeled according to the reported death dates. The maximal follow-up time was less than 13 years for those interviewed in 2004. The survival and follow-up time for each frailty index were listed in Table 2. The pattern of yearly mortality risks since 2004 interview was described with Kaplan-Meier survival function[31]. Because of the violation of the proportional hazard assumption of the Cox proportional model, discrete-time survival analysis was adopted[32]. Mortality risk associated with three frailty indices, bias variables, input or domain variables and alternative indices were estimated while controlling for sex, race/ethnicity, education, per capita income, and per capita wealth[32].

**Table 2:** Survival analysis for the comparison of predictive power.

	1. Functional Domains Model	2. Burden Model	3. Biologic Syndrome Model	Body Mass Index
**Numbers excluded for lack of race/ethnicity (n)**	1	1	0	0
**Numbers excluded for lack of survival status (n)**	87	44	9	331
**Sample sizes for survival analysis (n)**	11025	7668	1633	19419
**Mean follow-up time (years)**	7.46	6.94	7.7	8.14
**Mean survival time if died (years)**	4.93	4.8	5.47	5
**Mean follow-up time if survived (years)**	9.51	9.48	9.63	9.51
**Proportions of death**	0.443	0.54	0.462	0.489
**Log p values of frailty indices to predict mortality**	-287	-373	-63	-114
**Frailty indices are sums of significant mortality predictors**	+	+	+	+
**Numbers of input variables**	9	26	15	2
**Numbers of input variables significantly predicting mortality**	9	24	13	2
**Indices in continuous scales better predicting mortality than dichotomous ones**	+	+	+	+
**Input or domain variables better predicting mortality than indices**	+	+	+	
**AUC (95% CI) of continuous frailty indices from survival analysis**	0.741 (0.734 to 0.748)	0.731 (0.724 to 0.739)	0.766 (0.749 to 0.782))	0.585 (0.576 to 0.593)
**AUC (95% CI) of dichotomous frailty indices from survival analysis**	0.736 (0.729 to 0.743)	0.721 (0.713 to 0.728)	0.754 (0.736 to 0.771)	0.589 (0.581 to 0.597)
**AUC (95% CI) of domain variables from survival analysis**	0.744 (0.737 to 0.751)	0.752 (0.745 to 0.759)	0.768 (0.751 to 0.784)	
**AUC (95% CI) of bias variables from survival analysis**	0.73 (0.723 to 0.737)	0.701 (0.692 to 0.709)	0.759 (0.742 to 0.776)	0.547 (0.539 to 0.555)
**AUC (95% CI) of input variables from survival analysis**	0.758 (0.751 to 0.765)	0.758 (0.751 to 0.766)	0.778 (0.762 to 0.794)	0.585 (0.576 to 0.593)
**Numbers of variables to generate alternative indices**	70	70	72	
**Numbers of all alternative indices**	814385	814385	1028775	
**Numbers of significant alternative indices**	9827	11891	44648	
**Numbers of alternative indices with lower p values**	6865	8135	30018	
**Numbers of alternative indices with better residual deviances**	202932	31078	2849	
**Input variables of best performing 4-item indices**	1) Impaired mobility; 2) Impaired cognition based on performance-based scores or proxy assessment; 3) Summary scores of physical activities; 4) Dummy: Problems with bathing	1) Impaired mobility; 2) Impaired cognition based on performance-based scores or proxy assessment; 3) Summary scores of physical activities; 4) Dummy: Problems with bathing	1) Congestive heart failure; 2) Impaired mobility; 3) Slowness: Time to walk 8 ft, converted to time to walk 15 ft. Cutoff criteria according to sex and height remain the same; 4) Summary scores of physical activities	

Note: AIC = Akaike Information Criterion; AUC = area under curve; HRS = Health and Retirement Study. Body Mass Index (BMI) is dichotomized into two categories, overweight/obesity (BMI>25) and unerweight/normal.

The predictive power of three frailty indices in continuous scales or dichotomous statuses regarding mortality was compared with respective sets of own domain, bias and input variables, while above-mentioned individual characteristics were controlled for. The AUC of ROC curves with 95% confidence intervals (CIs)[30] of the models were shown for the comparison of predictive power.

### Search for alternative frailty indices

Indices were defined as composite measures that were the sum of more than one variable assigned with equal weights[16, 33]. There were 44 input variables, 19 derived variables to generate domain variables, and 9 domain variables, 72 in total. These variables related to the creation of frailty indices were used to compose new empirical frailty indices alternative to the three frailty indices. Because there were a large number of combinations of the variables to create indices (2^72^–73), it was not likely to compute the predictive power of all possible indices within a reasonable time period. Therefore we use all combinations of any four input or domain variables, 1028790 in total, to explore the ranges of plausible and achievable predictive power in terms of p values of alternative indices, model AIC values, and residual deviances relative to null models[28]. P values less than 0.05 were considered statistically significant. All statistical analyses and data processing were conducted with R (v3.31) [34] and RStudio (v1.0.44) [35].

## Results

The frailty indices in Cigolle et al. (2009)[2] and our temporary ones were considered comparable because of the acceptable differences in samples sizes and weighted prevalence rates in Table 1. The sample sizes for the Functional Domains Model were the same in Cigolle et al. (2009) and this study. We identified six and 15 fewer subjects in this study for the Burden Model and the Biologic Syndrome Model respectively, compared to Cigolle et al. (2009). The prevalence rates of the three models in Cigolle et al. (2009), 29%, 32% and 11% respectively, were similar to our estimates.

The frailty statuses defined by three models were prevalent among the elderly, 27.5%, 44.8% and 16.8% among those aged 65, 70 and 65 years and over respectively in Table 1. However, frailty was also prevalent among those younger than the age criteria, 13.8%, 23.9% and 10% respectively. The minimal age of the participants were 24 years for the three indices.

### Relationships with input variables

**Fig 3:**
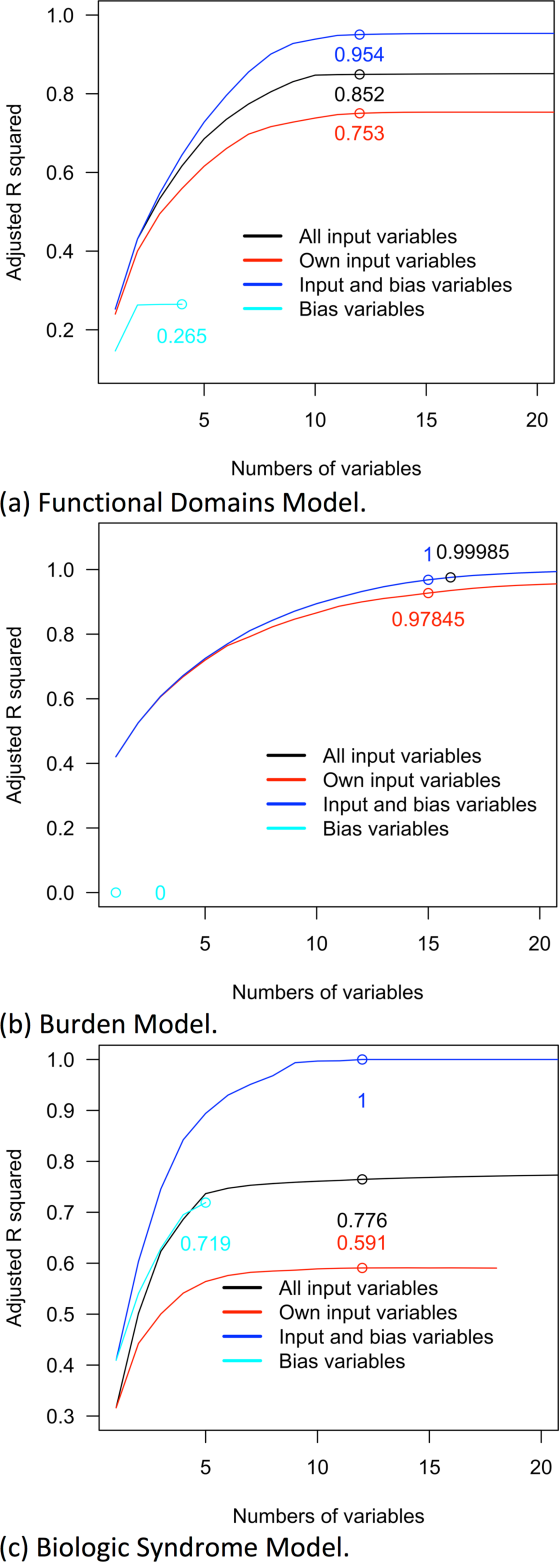
R squared curves derived from the approximation of three frailty indices with input and bias variables. (a) Functional Domains Model. (b) Burden Model. (c) Biologic Syndrome Model. Note: the lines showing the values of adjusted R squared by numbers of input or bias variables. Adjusted R squared was generated with forward-selection regression models with eligible participants and frailty indices created with imputed data. The locations of the circles might not be on the peak points and were labelled for the ease of interpretation.

None of the three frailty indices in continuous scales could be fully explained by their own input variables (see statistics in Table 1). This suggested at least 24.7%, 2.2% and 41.6% of the respective index variances originated from the bias due to data processing and was unrelated to input variables. In contrast, 0.5% of the BMI variance could not be explained by height, weight and their interaction term. With more input variables in forward-selection regression models, the lines of adjusted R squared approached the top of the charts in Fig 3. The three indices could be fully explained, R squared equal to 1.0, only when both input and bias variables were included as independent variables. If only bias variables were retained to approximate frailty indices, the proportions of variances explained solely by bias alone ranged from 0.3%, the Burden Model, to 73.7%, the Biological Syndrome Model.

None of the three frailty statuses could be fully explained by the input variables. The frailty statuses, yes or no, defined by three models was not interpreted linearly. The frailty statuses defined by the three indices could be well approximated with 27, 54 and 29 input variables respectively with the AUCs covering more than 96.5% of the plot area (Table 1). However, the bias variables could be related to the AUCs covering 75.5%, 44.0% and 96.8% of the plot area for three respective indices. The bias variables predicted the frailty status in the Biological Syndrome Model better than input variables (see S3 Appendix for the ROC curves).

Complex indices could be simplified by having less numbers of input variables if full approximation was not required. The Burden Model in continuous scale that required 25 input variables could be approached by less numbers of variables, 11, 14 and 20 variables to explain more than 90%, 95% and 99% of variances respectively.

### Interpretation of frailty indices and statuses

The proportions of the frailty index variances that could be explained by their own input variables were 75.3%, 97.8% and 58.4% respectively. The four leading variables could explain 55.9%, 66.6% and 54.6% of index variances respectively. The four leading variables explaining the frailty index in the Functional Domain Model were related to physical functioning, cognitive impairment and poor hearing in Table 1. The four leading variables for the Burden Model were related to stroke, depression, impaired mobility and urinary incontinence. The leading ones for the Biological Syndrome Model were feeling everything an effort, lack of motivation, less physical activities, and poor grip strength.

### Predictive power of frailty indices on mortality

There were less than 90 participants excluded for the lack of information on race/ethnicity and survival statuses for three frailty indices. The sample sizes for survival analysis were 11025, 7668, and 1633 respectively in Table 2. The mean follow-up time in years ranged from 4.8 to 5.47. The survival functions by sex or race/ethnicity were shown in S4 Appendix.

There were common patterns in the order of mortality predictive power (see S1 Appendix for detailed statistics). Overall, the input variables best predicted mortality probability among the interviewees eligible to the three frailty models. The input or domain variables both better predicted mortality risks than frailty indices. Continuous frailty indices better predicted mortality than dichotomous frailty statuses. Unexpectedly, the bias variables of the Biological Syndrome Model could predict mortality better than dichotomous frailty status.

### Survival analysis and alternative frailty indices

There were 1028790 combinations of any four variables out of 72 input or domain variables. Because not all variables were measured among the interviewees eligible for the three respective frailty models, the numbers of applicable combinations were less than the maximal value (see Table 2 for details and S5 Appendix for the ROC curves). Out of 814385, 814385, and 1028775 alternative frailty indices, there were 9827, 11891 and 44648 ones significantly predicting mortality for respective models. Among significant alternative indices, there were 6865, 8135, and 30018 ones better predict mortality than the three respective indices in terms of p values (Table 2). Impaired mobility and summary scores of physical activities were the common variables in the three alternative indices that best predicted mortality in respective populations.

## Discussion

The three frailty indices are created based on theories and assumptions. After carefully examining the indices and their relationships with input variables, we identify several issues and problematic assumptions. Using BMI as a comparison, we think these issues are not inevitable and can be avoided with caution and sound data practices. The first issue is that there is bias originated from data processing and manipulation. The three frailty indices consist of information from the input variables and a varying degree of bias or noise generated purely due to data manipulation. The magnitude of bias in the Biological Syndrome Model is so large that the bias variables explain more than 76% of the index variance.

Second, the idea of frailty is not easy to understand and the three frailty indices have never been interpreted with input variables to the best of our knowledge. Third, the frailty indices are not well connected with the theories due to three reasons: the data distortion we mentioned in the Introduction, redundant variables, and bias generated from data manipulation. Conceptually, the frailty theories are the basis to select the input variables for index creation. The input variables represent the idea that the indices aim to capture. Ideally, the theory-based input variables should fully support the frailty indices, 100% index variances explained by the input variables (see the Introduction for the description of the input variables and Table 1 for proportions of variances explained). However, only part of the index variances can be explained by input variables. For the Burden Model, it requires far fewer theory-based input variables to interpret most of the index variances (see Table 1 for the numbers of input variables).

Based on our analysis, it is uncertain whether the frailty indices created are the same as those that researchers intended to generate. For example, stroke, depression, impaired mobility and urinary incontinence are the leading variables to explain the frailty index in the Burden Model that consists of 25 input variables. Based on the variance explained by these variables, this model defined by deficit accumulation seems to prefer conditions associated with these four. For the other two models, we do not find evidence that suggests the four leading variables that best explain the frailty indices in Table 1 being emphasized in the theories or variable selection process. It is unclear whether the generated indices are reflective of the theories or the authors’ intention.

Fourth, the frailty indices or statuses are not optimal predictors. The frailty statuses defined by the three models have been commonly used to predict outcomes. The predictive power of frailty statuses regarding major outcomes, especially mortality, has become a rationale for frailty[4, 8, 18]. However, the input or domain variables predict mortality better than frailty indices or statuses. Frailty indices also predict mortality better than frailty statuses. One reason for the reduced predictive power is that linking variables through an index is assuming these variables have equal regression coefficients towards any outcomes[16]. This is a restrictive assumption that in most cases predictive power will be compromised[16]. The other reason may be that the transition from non-frail to frail, especially the pre-frail stage, may be underestimated for the role of mortality prediction. We strongly advise using frailty indices or statuses to predict outcomes only after the input and domain variables have been assessed for predictability. In consideration of the bias generated due to the categorization of continuous variables[21], we recommend using categorized variables for descriptive purposes and using continuous variables and input variables for outcome prediction. Lastly, there are numerous alternative indices that better predict mortality. There are variables common to the best performing four-item indices, impaired mobility and summary scores of physical activities. These two variables seem to resemble the physical functioning domain in the Functional Domains Model, some deficits in the Burden models, and low energy expenditure domain in the Biological Syndrome Model. These two variables can be considered if frailty researchers aim to develop alternative evidence-based indices.

In addition to the above-mentioned issues regarding index creation and data processing, there are problematic assumptions related to the use and creation of frailty indices. First, frailty has been commonly used as a state with clear cut-off values[2]. The idea of pre-frail stage originally proposed[4] has not been widely used in studies[2, 7–10]. From our perspective, frailty may be better regarded as a continuum. Dichotomizing frailty index involves bias that is difficult to explain and interpret (Fig 2). Second, it is implicitly assumed that there is a possible range of frailty prevalence. However, there is a lack of consensus regarding the “optimal” ranges of frailty prevalence. It is argued that prevalent symptoms among the elderly cannot be counted as a frailty deficit in the Burden Model, because these variables “saturate too early” based on their theory[19]. In contrast, the Biologic Syndrome Model assumes that each of two frailty criteria should be applicable to at least 20% of the population[2]. The conflicting views of how prevalent frailty remain to be resolved. Third, the age criteria are another assumption about when frailty should occur. Frailty is often assumed or proposed to be a geriatric syndrome[2–4, 19, 36]. Three frailty models have age eligibility criteria set at 65 or 70 years of age[2]. However, we and other researchers have found that frailty is also prevalent in younger populations[36][37]. Previous research has focused on searching for frailty traits among the elder and identified measures that might be sensitive among the elderly and unspecific to the other age groups. The concept of frailty may not be precise enough and four other frailty indices are also unspecific to the elderly[38].

Fourth, it is assumed that input variables are similarly important and equal weights are applied. This assumption is not fully disclosed or executed. Although all input variables contribute to the final indices, the relationships between input variables and indices are distorted by data manipulation and data redundancy. The bias introduced by categorization and top censoring can distort the information contributed and thus inflate or deflate the importance of certain input variables. Besides data manipulation, another reason is that the sum of multiple highly correlated variables may not be more informative than any one of them. Without explicitly designing variable selection criteria and weighting scheme to search for unique sources of information to represent the desired frailty domains[16, 28], this problem can worsen if more variables that provide overlapping information are added together. Lastly, the biology of frailty and the measurement in the populations are not well connected. For example, lung disease is one of the criteria for a 70-item frailty index[3]. However, lung function has not been directly linked to the biology of frailty[11].

### Implication on clinical trials and reproducible research

It is time to better discuss frailty and clarify its definition as an intervention target or a proxy measure of patient status. By searching PubMed database and ClinicalTrials.gov with the term “frailty”, there are respectively 6981 articles published and 317 clinical trials registered before the end of 2016. New outcomes that cannot be easily interpreted can be false targets that lead to interventions without benefits or trials causing harm. A similar example is the creation of metabolic syndrome that is confirmed with three out of five diagnostic criteria[39]. This diagnosis is later found to bear little benefits to predict two patient outcomes: diabetes and cardiovascular disease[40, 41]. With the problems identified in the three frailty indices and the recent advances in index mining[16], we are concerned about the role of bias and whether the frailty indices merely represent data illusion. To improve the science of index mining, we propose a draft guide for researchers to create new indices that are interpretable and useful (S6 Appendix).

We also notice that there are still obstacles for reproducible research. Based on our experiences, we find that there is no sufficient information in the publications to fully reproduce the frailty indices for several reasons. First, each of the research groups of three frailty indices use specialized data sets that few have the access to. We are fortunate to build on the works by Cigolle et al. (2009) that adopted an open data set to compare different frailty indices. Second, there was no requirement for the authors to archive the programming codes and information on variables. Even with numerous publications by three research groups and Cigolle et al.’s efforts to reproduce, many of the definitions of the input variables remain vague and arbitrary. We think the lack of transparency and reproducibility is an important issue for researchers considering adopting frailty indices as an outcome in clinical trials.

### Limitations

Whether the frailty indices of Cigolle et al. (2009) are exactly reproduced is uncertain, especially for the Burden Model. This is because the information on variable names, missing data imputation and ineligibility is not fully disclosed or revealed. Although the sample sizes of the eligible individuals of three respective indices are similar or the same as those published, there are slight discrepancies in the prevalence of three frailty indices. However, this limitation does not invalidate the results.

## Conclusion

The frailty statuses defined by the three models are syndromes not unique to the elderly. The three frailty indices we reviewed consist of a varying degree of bias introduced due to data processing and manipulation. The frailty indices or the frailty statuses cannot be fully explained by their own input variables. The frailty statuses do not predict mortality better than the continuous indices. The input and domain variables predict mortality better than the frailty indices. The bias introduced by the Biological Syndrome Model predicts mortality better than the frailty status. There are at least 6865 alternative 4-item indices that better predict mortality than the frailty indices. It is recommended to use the frailty indices with caution and understand the sources of bias. We are developing new algorithms to uncover new and useful indices and developing a guide to report new indices that can be reproduced and interpreted with input variables.

## Supporting information

S1 Appendix Problems of the three frailty indices identified after literature review and statistical analysis. (DOCX)

S2 Appendix Characteristics of input variables of three frailty indices. (DOCX)

S3 Appendix ROC curves derived from the approximation of frailty indices with input and bias variables. (DOCX)

S4 Appendix Survival curves for the Health and Retirement Study interviewees. (DOCX)

S5 Appendix ROC curves derived from approximating the three frailty statuses with input or bias variables. (DOCX)

S6 Appendix A draft guide to mine index and report mining process. (DOCX)
