# Supplementary material for: Index or illusion: The case of frailty indices in the Health and Retirement Study
Source: PLoS One. 2018 Jul 18;13(7):e0197859. doi: 10.1371/journal.pone.0197859 (PMC6051600; doi:10.1371/journal.pone.0197859)
Supplement: S1 Appendix — (DOCX) [file pone.0197859.s001.docx]

**Appendix 1**. Problems of the three frailty index identified after literature review and statistical analysis.

|  | Problems identified after literature review |  |  |  |  |
| --- | --- | --- | --- | --- | --- |
|  |  | 1. Functional Domains Model | 2. Burden Model | 3. Biologic Syndrome Model | Body Mass Index |
| A. implicit assumptions imposed by the index criteria |  |  |  |  |  |
|  | 1. frailty is a state not commonly described as a continuum; pre-frail stage often ignored | + | + | + |  |
|  | 2. common symptoms cannot be counted as an attribute to frailty, while cut-off prevalence rates unknown |  | + |  |  |
|  | 3. common symptoms need to be counted as attributes to frailty: at least 20% of eligible populations qualified for frailty attributes |  |  | + |  |
|  | 4. age criteria as an assumption about when frailty should occur | + | + | + |  |
| B. unclear weighting schemes for each candidate domains of frailty |  |  |  |  |  |
|  | 5. lack of criteria to exclude highly correlated or duplicate measures; over-emphasizing particular functional domains by including highly correlated variables |  | + |  |  |
|  | 6. assigning equal weights that may not be optimal for outcome prediction | + | + | + |  |
|  | 7. "other medical history" as a deficit; overemphasis of existing domains possible |  | + |  |  |
| C. data processing that may be prone to bias or not based on evidence |  |  |  |  |  |
|  | 8. bias introduced because continuous variables are often regrouped to discrete variables | + | + | + |  |
|  | 9. top-censoring of the sum of two or more variables | + |  | + |  |
|  | 10. ordinal variables taken as interval variables and scaled within the range of zero and one by division |  | + |  |  |
| D. survey design not properly controlled |  |  |  |  |  |
|  | 11. applying unweighted percentiles to derive weighted statistics |  |  | + |  |
| E. disconnection between the biology of frailty and the measurement |  |  |  |  |  |
|  | 12. biology of frailty and the measurement in the populations not well connected |  | + |  |  |
|  |  |  |  |  |  |
|  | Problems identified after index replication and analysis |  |  |  |  |
|  |  | 1. Functional Domains Model | 2. Burden Model | 3. Biologic Syndrome Model | Body Mass Index |
| F. published frailty indices not fully reproducible |  |  |  |  |  |
|  | 13. unclear missing value processing | + | + | + |  |
|  | 14. input variables not revealed |  | + |  |  |
|  | Basic characteristics of frailty indices |  |  |  |  |
|  | Numbers of domains or items | 4 | 24 (representing 30 of original 70 items; 38 used in Cigolle et al. (2009))) | 5 | 1 |
|  | Numbers of variables required | 9 | 25 | 10 | 2 |
|  | Numbers of sources of bias due to data manipulation | 4 | 1 | 5 | 1 |
|  | Sample sizes in Cigolle et al. (2009) | 11,113 | 7,719 | 1,657 |  |
|  | Inclusion criteria in Cigolle et al. (2009) | Adults aged 65 and older | Adults aged 70 and older | Adults aged 65 and older who completed the performance measures and did not have stroke, depression, or moderate to severe cognitive impairment |  |
|  | Weighted frailty prevalence in Cigolle et al. (2009) (%) | 29% | 32% | 11% |  |
|  | Samples sizes retrieved in this study (n) | 11113 | 7713 | 1642 | 19750 |
|  | Weighted frailty prevalence in this study (%) | 26.77% | 42.30% or 27.62% (divided by 24 variables or 30 items represented) | 11.16% |  |
|  | Complete cases (n) | 9314 | 6635 | 1429 | 1662 |
|  | Proportions of complete cases | 0.8381 | 0.8602 | 0.8703 | 0.0842 |
|  | Weighted frailty prevalence based on imputed data | 26.79% | 43.51% or 29.30% (divided by 24 variables or 30 items represented) | 10.62% |  |
| G. frailty indices created are not the same as those intended or desired |  |  |  |  |  |
|  | Age eligibility | 65 years and over | 70 years and over | 65 years and over |  |
|  | Unweighted prevalence of those meeting age criteria | 0.275 | 0.446 | 0.167 |  |
|  | Unweighted prevalence of those younger than age criteria (minimal age: 24 years) | 0.139 | 0.240 | 0.096 |  |
|  | 16. continuous frailty indices not fully explained by original input variables | + | + | + | + |
|  | Proportions of variances not explained by input variables | 0.1475 | 0.00015 | 0.2237 | 0.0058 |
|  | 17. frailty statues could not be explained by original input variables | + | + | + | + |
|  | 18. bias or noise of various magnitudes existing in three frailty indices | + | + | + | + |
|  | Numbers of variables used to approximate continuous frailty indices | 70 | 72 | 66 | 2 |
|  | Numbers of sources of bias due to data manipulation | 4 | 1 | 5 | 1 |
|  | Maximal proportions of variances of continuous frailty indices explained by own input variables (R squared) | 0.753 | 0.978 | 0.591 | 0.994 |
|  | Numbers of own input variables required for maximal R squared | 17 | 48 | 14 | 3 |
|  | Maximal proportions of variances of continuous frailty indices explained by all input variables (R squared) | 0.852 | 0.99985 | 0.776 | 0.994 |
|  | Numbers of all input variables required for maximal R squared | 55 | 56 | 42 | 3 |
|  | Maximal proportions of variances of continuous frailty indices explained by bias along (R squared) | 0.265 | <0.001 | 0.719 | 0.006 |
|  | Numbers of bias sources for maximal R squared | 4 | 1 | 5 | 1 |
|  | Numbers of variables used to approximate dichotomous frailty status | 49 | 49 | 45 | 2 |
|  | Numbers of variables to achieve maximal AIC | 27 | 54 | 29 | 3 |
|  | AUC of best fit models (95% CIs) with input variables | 0.973 (0.971 to 0.976) | 0.967 (0.963 to 0.97) | 0.965 (0.955 to 0.975) | 1 (1 to 1) |
|  | AUC of best fit models (95% CIs) with bias variables | 0.755 (0.743 to 0.767) | 0.44 (0.426 to 0.455) | 0.968 (0.959 to 0.976) | 0.522 (0.513 to 0.531 ) |
| H. complex frailty index could be simplified |  |  |  |  |  |
|  | 19. complex frailty index could be simplified |  | + |  |  |
|  | 20. number of input variables to explain 90% of the variance of continuous indices |  | 11 |  |  |
|  | 21. number of input variables to explain 95% of the variance of continuous indices |  | 14 |  |  |
|  | 22. number of input variables to explain 99% of the variance of continuous indices |  | 20 |  |  |
|  |  |  |  |  |  |
|  | Survival analysis for the comparison of predictive power |  |  |  |  |
|  |  | 1. Functional Domains Model | 2. Burden Model | 3. Biologic Syndrome Model | Body Mass Index |
| I. arbitrary constraint on the regression coefficients of input or domain variables to predict mortality |  |  |  |  |  |
|  | 23. similar regression coefficients for input or domain variables not optimal to predict mortality | + | + | + |  |
|  | Regression coefficients of original or domain variables not the same as those derived from indices | + | + | + |  |
|  | 24. not all input variables necessary to better predict mortality | + | + | + |  |
| J. relatively poor predictive power regarding mortality |  |  |  |  |  |
|  | Discrete-time survival analysis |  |  |  |  |
|  | Numbers excluded for lack of race/ethnicity (n) | 1 | 1 | 0 | 0 |
|  | Numbers excluded for lack of survival status (n) | 87 | 44 | 9 | 331 |
|  | Sample sizes for survival analysis (n) | 11025 | 7668 | 1633 | 19419 |
|  | Mean follow-up time (years) | 7.46 | 6.94 | 7.7 | 8.14 |
|  | Mean survival time if died (years) | 4.93 | 4.8 | 5.47 | 5 |
|  | Mean follow-up time if survived (years) | 9.51 | 9.48 | 9.63 | 9.51 |
|  | Proportions of death | 0.443 | 0.54 | 0.462 | 0.489 |
|  | Log p values of frailty indices to predict mortality | -287 | -373 | -63 | -114 |
|  | 25. frailty indices are sums of significant mortality predictors | + | + | + | + |
|  | Numbers of input variables | 9 | 26 | 15 | 2 |
|  | Numbers of input variables significantly predicting mortality | 9 | 24 | 13 | 2 |
|  | 26. indices in continuous scales better predicting mortality than dichotomous ones | + | + | + | + |
|  | 27. input or domain variables better predicting mortality than indices | + | + | + |  |
|  | AUC (95% CI) of continuous frailty indices from survival analysis | 0.741 (0.734 to 0.748) | 0.731 (0.724 to 0.739) | 0.766 (0.749 to 0.782) | 0.585 (0.576 to 0.593) |
|  | AUC (95% CI) of dichotomous frailty indices from survival analysis | 0.736 (0.729 to 0.743) | 0.721 (0.713 to 0.728) | 0.754 (0.736 to 0.771) | 0.589 (0.581 to 0.597) |
|  | AUC (95% CI) of domain variables from survival analysis | 0.744 (0.737 to 0.751) | 0.752 (0.745 to 0.759) | 0.768 (0.751 to 0.784) |  |
|  | AUC (95% CI) of bias variables from survival analysis | 0.73 (0.723 to 0.737) | 0.701 (0.692 to 0.709) | 0.759 (0.742 to 0.776) | 0.547 (0.539 to 0.555) |
|  | AUC (95% CI) of input variables from survival analysis | 0.758 (0.751 to 0.765) | 0.758 (0.751 to 0.766) | 0.778 (0.762 to 0.794) | 0.585 (0.576 to 0.593) |
