# Supplementary material for: Index or illusion: The case of frailty indices in the Health and Retirement Study
Source: PLoS One. 2018 Jul 18;13(7):e0197859. doi: 10.1371/journal.pone.0197859 (PMC6051600; doi:10.1371/journal.pone.0197859)
Supplement: S2 Appendix — (DOCX) [file pone.0197859.s002.docx]

Appendix 2. Characteristics of input variables of three frailty indices.

| Variable names | Imported from 2004 wave HRS data | Numbers of missing values among those eligible for the first frailty index | Proportions of missing values relative to those eligible for the first frailty index | Numbers of missing values among those eligible for the second frailty index | Proportions of missing values relative to those eligible for the second frailty index | Numbers of missing values among those eligible for the third frailty index | Proportions of missing values relative to those eligible for the third frailty index | Original RAND HRS variables | frail1 | frailcat1 | frail2 | frailcat2 | frail3 | frailcat3 | Variable definitions | All possible responses (if categorical variables) | Types of variables |
| --- | --- | --- | --- | --- | --- | --- | --- | --- | --- | --- | --- | --- | --- | --- | --- | --- | --- |
| r7depres |  | 1248 | 0.112 | 954 | 0.124 | 0 | 0.000 | TRUE | TRUE | TRUE | TRUE | TRUE |  |  | Feeling sad, blue, depressed | 0.no; 1.yes | factor |
| r7effort |  | 1254 | 0.113 | 959 | 0.124 | 1 | 0.001 | TRUE | TRUE | TRUE | TRUE | TRUE | TRUE | TRUE | (i) Felt that everything I did was an effort in last week. | 0.no; 1.yes | factor |
| r7sleepr |  | 1245 | 0.112 | 954 | 0.124 | 0 | 0.000 | TRUE | TRUE | TRUE | TRUE | TRUE | TRUE | TRUE | Sleep changes | 0.no; 1.yes | factor |
| r7going |  | 1264 | 0.114 | 969 | 0.126 | 1 | 0.001 | TRUE | TRUE | TRUE | TRUE | TRUE | TRUE | TRUE | (ii) Could not get going in last week. | 0.no; 1.yes | factor |
| r7vgactx |  | 10 | 0.001 | 9 | 0.001 | 3 | 0.002 | TRUE | TRUE | TRUE | TRUE | TRUE | TRUE | TRUE | Low energy expenditure: Frequency of three intensities of activity, lowest quintile (stratified according to sex) | 2.>1 per week; 3.1 per week; 5.never; 4.l-3 per mon; 1.every day | factor |
| r7mdactx |  | 9 | 0.001 | 5 | 0.001 | 1 | 0.001 | TRUE | TRUE | TRUE | TRUE | TRUE | TRUE | TRUE | Low energy expenditure: Frequency of three intensities of activity, lowest quintile (stratified according to sex) | 4.l-3 per mon; 2.>1 per week; 5.never; 3.1 per week; 1.every day | factor |
| r7ltactx |  | 6 | 0.001 | 3 | 0.000 | 1 | 0.001 | TRUE | TRUE | TRUE | TRUE | TRUE | TRUE | TRUE | Low energy expenditure: Frequency of three intensities of activity, lowest quintile (stratified according to sex) | 3.1 per week; 2.>1 per week; 5.never; 4.l-3 per mon; 1.every day | factor |
| r7bmi |  | 161 | 0.014 | 113 | 0.015 | 10 | 0.006 | TRUE | TRUE | TRUE | TRUE | TRUE | TRUE | TRUE | Weight loss: BMI o18.5 kg/m2 | | numeric |
| r7height |  | 18 | 0.002 | 17 | 0.002 | 1 | 0.001 | TRUE | TRUE | TRUE | TRUE | TRUE | TRUE | TRUE | Height in meters |  | numeric |
| r7weight |  | 143 | 0.013 | 96 | 0.012 | 9 | 0.005 | TRUE | TRUE | TRUE | TRUE | TRUE | TRUE | TRUE | Weight loss: Weight in wave 2002 minus weight in wave 2004 & gain weight for > 10% of weight in wave 2002 | | numeric |
| r7hibp |  | 20 | 0.002 | 18 | 0.002 | 1 | 0.001 | TRUE | TRUE | TRUE | TRUE | TRUE | TRUE | TRUE | Arterial hypertension | 0.no; 1.yes; 4.display previous record and no condition; 3.display previous record and has condition | factor |
| r7diabs |  | 79 | 0.007 | 50 | 0.006 | 0 | 0.000 | TRUE | TRUE | TRUE | TRUE | TRUE | TRUE | TRUE | History of diabetes mellitus | 0.no; 1.yes | factor |
| r7cancr |  | 10 | 0.001 | 5 | 0.001 | 0 | 0.000 | TRUE | TRUE | TRUE | TRUE | TRUE | TRUE | TRUE | Malignant disease | 0.no; 1.yes; 4.display previous record and no condition; 3.display previous record and has condition | factor |
| r7lung |  | 10 | 0.001 | 8 | 0.001 | 1 | 0.001 | TRUE | TRUE | TRUE | TRUE | TRUE | TRUE | TRUE | Lung problems or Respiratory problems | 0.no; 1.yes; 4.display previous record and no condition; 3.display previous record and has condition | factor |
| r7heart | TRUE | 15 | 0.001 | 14 | 0.002 | 1 | 0.001 | TRUE | TRUE | TRUE | TRUE | TRUE | TRUE | TRUE | Congestive heart failure | 1.yes; 0.no; 4.display previous record and no condition; 3.display previous record and has condition | factor |
| r7strok |  | 6 | 0.001 | 4 | 0.001 | 0 | 0.000 | TRUE | TRUE | TRUE | TRUE | TRUE | TRUE | TRUE | Cerebrovascular problems | 0.no; 1.yes; 4.display previous record and no condition; 2. Transient Ischemic Attack /possible stroke; 3.display previous record and has condition | factor |
| r7stroke |  | 12 | 0.001 | 6 | 0.001 | 0 | 0.000 | TRUE | TRUE | TRUE | TRUE | TRUE | TRUE | TRUE | History of stroke | 0.no; 1.yes | factor |
| r7stroks |  | 69 | 0.006 | 42 | 0.005 | 0 | 0.000 | TRUE | TRUE | TRUE | TRUE | TRUE |  |  |  | 0.no; 1.yes | factor |
| r7psych |  | 13 | 0.001 | 9 | 0.001 | 0 | 0.000 | TRUE | TRUE | TRUE | TRUE | TRUE | TRUE | TRUE | Depression (clinical impression) | 0.no; 1.yes; 4.display previous record and no condition; 3.display previous record and has condition | factor |
| r7psychs |  | 73 | 0.007 | 48 | 0.006 | 0 | 0.000 | TRUE | TRUE | TRUE | TRUE | TRUE | TRUE | TRUE | Changes in general mental functioning | 0.no; 1.yes | factor |
| r7arthr |  | 12 | 0.001 | 8 | 0.001 | 0 | 0.000 | TRUE | TRUE | TRUE | TRUE | TRUE | TRUE | TRUE | Other medical history | 0.no; 1.yes; 4.display previous record and no condition; 3.display previous record and has condition | factor |
| r7memrys |  | 123 | 0.011 | 75 | 0.010 | 0 | 0.000 | TRUE | TRUE | TRUE | TRUE | TRUE | TRUE | TRUE | Memory changes | 0.no; 1.yes | factor |
| r7dress |  | 7 | 0.001 | 6 | 0.001 | 0 | 0.000 | TRUE | TRUE | TRUE | TRUE | TRUE | TRUE | TRUE | Problems getting dressed | 0.no; 2.can't do; 1.yes; 9.don't do | factor |
| r7bath |  | 11 | 0.001 | 10 | 0.001 | 0 | 0.000 | TRUE | TRUE | TRUE | TRUE | TRUE | TRUE | TRUE | Problems with bathing | 0.no; 1.yes; 2.can't do; 9.don't do | factor |
| r7toilt |  | 11 | 0.001 | 10 | 0.001 | 0 | 0.000 | TRUE | TRUE | TRUE | TRUE | TRUE | TRUE | TRUE | Toileting problems | 0.no; 1.yes; 9.don't do; 2.can't do | factor |
| r7lift |  | 15 | 0.001 | 13 | 0.002 | 2 | 0.001 | TRUE | TRUE | TRUE | TRUE | TRUE | TRUE | TRUE | Physical functioning: difficulty lifting 10 pounds | 0.no; 2.can't do; 1.yes; 9.don't do | factor |
| r7mobila |  | 16 | 0.001 | 14 | 0.002 | 0 | 0.000 | TRUE | TRUE | TRUE | TRUE | TRUE | TRUE | TRUE | Impaired mobility | | integer |
| r7cogtot |  | 1257 | 0.113 | 941 | 0.122 | 0 | 0.000 | TRUE | TRUE | TRUE | TRUE | TRUE | TRUE | TRUE | History relevant to cognitive impairment or loss | | integer |
| r7fall | TRUE | 22 | 0.002 | 20 | 0.003 | 6 | 0.004 | TRUE | TRUE | TRUE | TRUE | TRUE | TRUE | TRUE | Falls |  | numeric |
| r7urine | TRUE | 48 | 0.004 | 37 | 0.005 | 1 | 0.001 | TRUE | TRUE | TRUE | TRUE | TRUE | TRUE | TRUE | Urinary incontinence | | numeric |
| r7eye | TRUE | 89 | 0.008 | 80 | 0.010 | 13 | 0.008 | TRUE | TRUE | TRUE | TRUE | TRUE | TRUE | TRUE | Sensory problems: Fair or poor eyesight despite use of corrective lenses | | character |
| r7hear | TRUE | 13 | 0.001 | 9 | 0.001 | 1 | 0.001 | TRUE | TRUE | TRUE | TRUE | TRUE | TRUE | TRUE | Sensory problems: fair or poor hearing despite use of hearing aides | | character |
| r7dizz | TRUE | 38 | 0.003 | 36 | 0.005 | 1 | 0.001 | TRUE | TRUE | TRUE | TRUE | TRUE | TRUE | TRUE | Physical functioning: Dizziness as persistent problem | | numeric |
| r7headac | TRUE | 25 | 0.002 | 22 | 0.003 | 0 | 0.000 | TRUE | TRUE | TRUE | TRUE | TRUE | TRUE | TRUE | Headache |  | numeric |
| r7tired | TRUE | 0 | 0.000 | 0 | 0.000 | 0 | 0.000 | TRUE | TRUE | TRUE | TRUE | TRUE |  |  | Tiredness all the time | | numeric |
| r7gripl | TRUE | 9191 | 0.827 | 6137 | 0.796 | 19 | 0.012 | TRUE | TRUE | TRUE | TRUE | TRUE | TRUE | TRUE | Grip strength, left hand |  | numeric |
| r7gripr | TRUE | 9204 | 0.828 | 6146 | 0.797 | 29 | 0.018 | TRUE | TRUE | TRUE | TRUE | TRUE | TRUE | TRUE | Grip strength, right hand |  | numeric |
| r7walkt | TRUE | 9245 | 0.832 | 6183 | 0.802 | 158 | 0.096 | TRUE | TRUE | TRUE | TRUE | TRUE | TRUE | TRUE | Slowness: Time to walk 8 ft, converted to time to walk 15 ft. Cut-off criteria according to sex and height remain the same | | numeric |
| r7muscle | TRUE | 4 | 0.000 | 3 | 0.000 | 1 | 0.001 | TRUE | TRUE | TRUE | TRUE | TRUE | TRUE | TRUE | Musculoskeletal problems | | numeric |
| r7memopr | TRUE | 37 | 0.003 | 11 | 0.001 | 0 | 0.000 | TRUE | TRUE | TRUE | TRUE | TRUE |  |  | Proxy memory rating | | character |
| r7seizure | TRUE | 0 | 0.000 | 0 | 0.000 | 0 | 0.000 | TRUE |  |  |  |  |  |  | Seizures, generalized | | numeric |
| r7grip |  | 9230 | 0.831 | 6169 | 0.800 | 48 | 0.029 | TRUE | TRUE | TRUE | TRUE | TRUE | TRUE | TRUE | Weakness: Grip strength: Weakest 20% (stratified according to sex and BMI) | | numeric |
| r7wchange | | 571 | 0.051 | 371 | 0.048 | 37 | 0.023 | TRUE | TRUE | TRUE | TRUE | TRUE | TRUE | TRUE | Weight in wave 2002 minus weight in wave 2004 (%) | | numeric |
| r7underw |  | 161 | 0.014 | 113 | 0.015 | 10 | 0.006 | TRUE | TRUE | TRUE | TRUE | TRUE | TRUE | TRUE | Underweight in wave 2004 (%) | | logical |
| r7fall_cat2 | TRUE | 22 | 0.002 | 20 | 0.003 | 6 | 0.004 | TRUE | TRUE |  | TRUE |  | TRUE |  | More than 2 falls | | numeric |
| r7cogimpair | | 37 | 0.003 | 11 | 0.001 | 0 | 0.000 | TRUE | TRUE | TRUE | TRUE | TRUE | TRUE | TRUE | Impaired cognition based on performance-based scores or proxy assessment | | numeric |
| r7frail1_1 |  | 0 | 0.000 | 0 | 0.000 | 0 | 0.000 | TRUE |  |  | TRUE |  | TRUE |  | Dizziness as persistent problem, >=2 falls in previous 2 years, or difficulty lifting 10 pounds | | numeric |
| r7frail1_2 |  | 0 | 0.000 | 0 | 0.000 | 0 | 0.000 | TRUE |  |  | TRUE |  | TRUE |  | Weight in wave 2002 minus weight in wave 2004 > 10% of weight in wave 2002 or body mass index < 18.5 kg/m2 | | numeric |
| r7frail1_3 |  | 0 | 0.000 | 0 | 0.000 | 0 | 0.000 | TRUE |  |  |  |  | TRUE |  | Mild to severe cognitive impairment on performance-based measure or according to proxy and interviewer rating | | numeric |
| r7frail1_4 |  | 0 | 0.000 | 0 | 0.000 | 0 | 0.000 | TRUE |  |  |  |  | TRUE |  | Fair or poor eyesight despite use of corrective lenses or fair or poor hearing despite use of hearing aides | | numeric |
| r7actsum |  | 20 | 0.002 | 13 | 0.002 | 4 | 0.002 | TRUE | TRUE | TRUE | TRUE | TRUE | TRUE | TRUE | Summary scores of physical activities | | numeric |
| r7walkt15 | TRUE | 9245 | 0.832 | 6183 | 0.802 | 158 | 0.096 | TRUE | TRUE | TRUE | TRUE | TRUE | TRUE | TRUE | Time to walk 15 feet | | numeric |
| r7frail3_2 |  | 6547 | 0.589 | 4321 | 0.560 | 0 | 0.000 | TRUE | TRUE |  | TRUE |  |  |  | Yes to either of two CES-D items: (i) Felt that everything I did was an effort in last week. (ii) Could not get going in last week. | | numeric |
| r7frail3_3 |  | 9471 | 0.852 | 6377 | 0.827 | 0 | 0.000 | TRUE | TRUE |  | TRUE |  |  |  | Frequency of three intensities of activity, lowest quintile (stratified according to sex) | | numeric |
| r7frail3_4 |  | 9247 | 0.832 | 6172 | 0.800 | 0 | 0.000 | TRUE | TRUE |  | TRUE |  |  |  | Time to walk 8 ft, converted to time to walk 15 ft. Cut-off criteria according to sex and height remain the same | | numeric |
| r7frail3_5 |  | 9471 | 0.852 | 6377 | 0.827 | 0 | 0.000 | TRUE | TRUE |  | TRUE |  | TRUE |  | Grip strength: Weakest 20% (stratified according to sex and BMI) | | numeric |
| r7fall_cat1 | TRUE | 22 | 0.002 | 20 | 0.003 | 6 | 0.004 | TRUE | TRUE |  | TRUE |  | TRUE |  | More than 1 falls | | numeric |
| r7dresscat |  | 7 | 0.001 | 6 | 0.001 | 0 | 0.000 | TRUE | TRUE |  | TRUE |  | TRUE |  | Dummy: problem getting dressed | | numeric |
| r7bathcat |  | 11 | 0.001 | 10 | 0.001 | 0 | 0.000 | TRUE | TRUE |  | TRUE |  | TRUE |  | Dummy: Problems with bathing | | numeric |
| r7toiltcat |  | 11 | 0.001 | 10 | 0.001 | 0 | 0.000 | TRUE | TRUE |  | TRUE |  | TRUE |  | Dummy: Toileting problems | | numeric |
| r7deprescat | | 1248 | 0.112 | 954 | 0.124 | 0 | 0.000 | TRUE | TRUE |  | TRUE |  |  |  | Dummy: Feeling sad, blue, depressed | | numeric |
| r7psychcat | | 13 | 0.001 | 9 | 0.001 | 0 | 0.000 | TRUE | TRUE |  | TRUE |  | TRUE |  | Dummy: Depression | | numeric |
| r7sleeprcat | | 1245 | 0.112 | 954 | 0.124 | 0 | 0.000 | TRUE | TRUE |  | TRUE |  | TRUE |  | Dummy: Sleep changes | | numeric |
| r7memryscat | | 123 | 0.011 | 75 | 0.010 | 0 | 0.000 | TRUE | TRUE |  | TRUE |  | TRUE |  | Dummy: Memory changes | | numeric |
| r7psychscat | | 73 | 0.007 | 48 | 0.006 | 0 | 0.000 | TRUE | TRUE |  | TRUE |  | TRUE |  | Dummy: Changes in general mental functioning | | numeric |
| r7strokcat |  | 6 | 0.001 | 4 | 0.001 | 0 | 0.000 | TRUE | TRUE |  | TRUE |  | TRUE |  | Dummy: Cerebrovascular problems | | numeric |
| r7strokecat | | 12 | 0.001 | 6 | 0.001 | 0 | 0.000 | TRUE | TRUE |  | TRUE |  | TRUE |  | Dummy: History of stroke | | numeric |
| r7diabscat |  | 79 | 0.007 | 50 | 0.006 | 0 | 0.000 | TRUE | TRUE |  | TRUE |  | TRUE |  | Dummy: History of diabetes mellitus | | numeric |
| r7heartcat | TRUE | 15 | 0.001 | 14 | 0.002 | 1 | 0.001 | TRUE | TRUE |  | TRUE |  | TRUE |  | Dummy: Cardiac problems | | numeric |
| r7lungcat |  | 10 | 0.001 | 8 | 0.001 | 1 | 0.001 | TRUE | TRUE |  | TRUE |  | TRUE |  | Dummy: Lung problems | | numeric |
| r7cancrcat | | 10 | 0.001 | 5 | 0.001 | 0 | 0.000 | TRUE | TRUE |  | TRUE |  | TRUE |  | Dummy: Malignant disease | | numeric |
| r7arthrcat |  | 12 | 0.001 | 8 | 0.001 | 0 | 0.000 | TRUE | TRUE |  | TRUE |  | TRUE |  | Dummy: Other medical history | | numeric |

Note: BMI: body mass index, CES-D = Center for Epidemiological Studies-Depression, frail1 = frailty index of the Functional Domain model proposed by Strawbridge et al. (1998), frailcat1 = frailty status according to the Functional Domain model proposed by Strawbridge et al. (1998), frail2 = frailty index of the Burden model by Rockwood et al. (2007), frailcat2 = frailty status according to the Burden model by Rockwood et al. (2007), frail3 = frailty index of the Biological Syndrome model by Fried et al. (2004), frailcat3 = frailty status according to the Biological Syndrome model by Fried et al. (2004), ft = feet, HRS: Health and Retirement Study, RAND = RAND Corporation
