# Supplementary material for: Index or illusion: The case of frailty indices in the Health and Retirement Study
Source: PLoS One. 2018 Jul 18;13(7):e0197859. doi: 10.1371/journal.pone.0197859 (PMC6051600; doi:10.1371/journal.pone.0197859)
Supplement: S3 Appendix — (DOCX) [file pone.0197859.s003.docx]

Appendix 3. ROC curves derived from the approximation of frailty indices with input and bias variables.


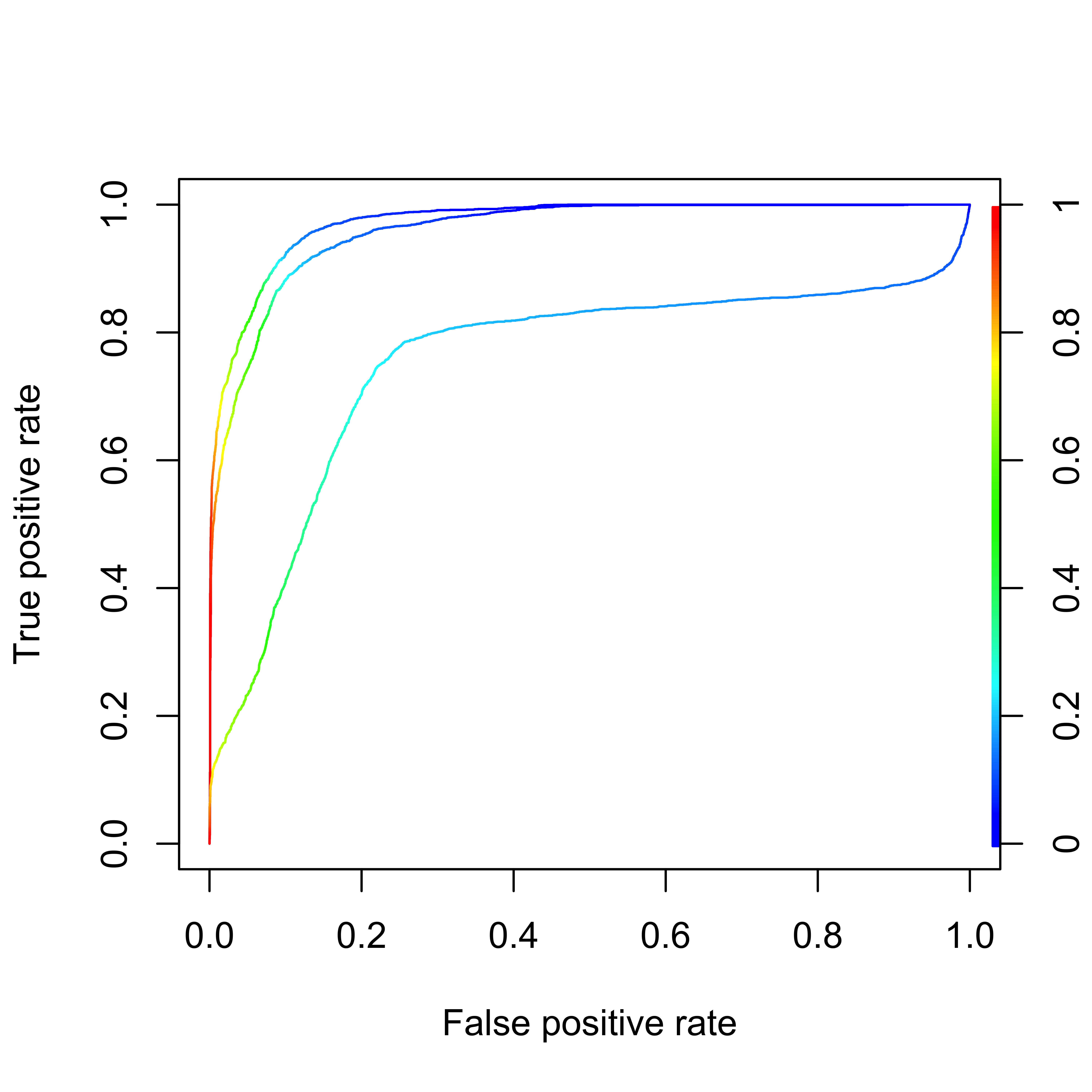


(a) Functional Domains Model.


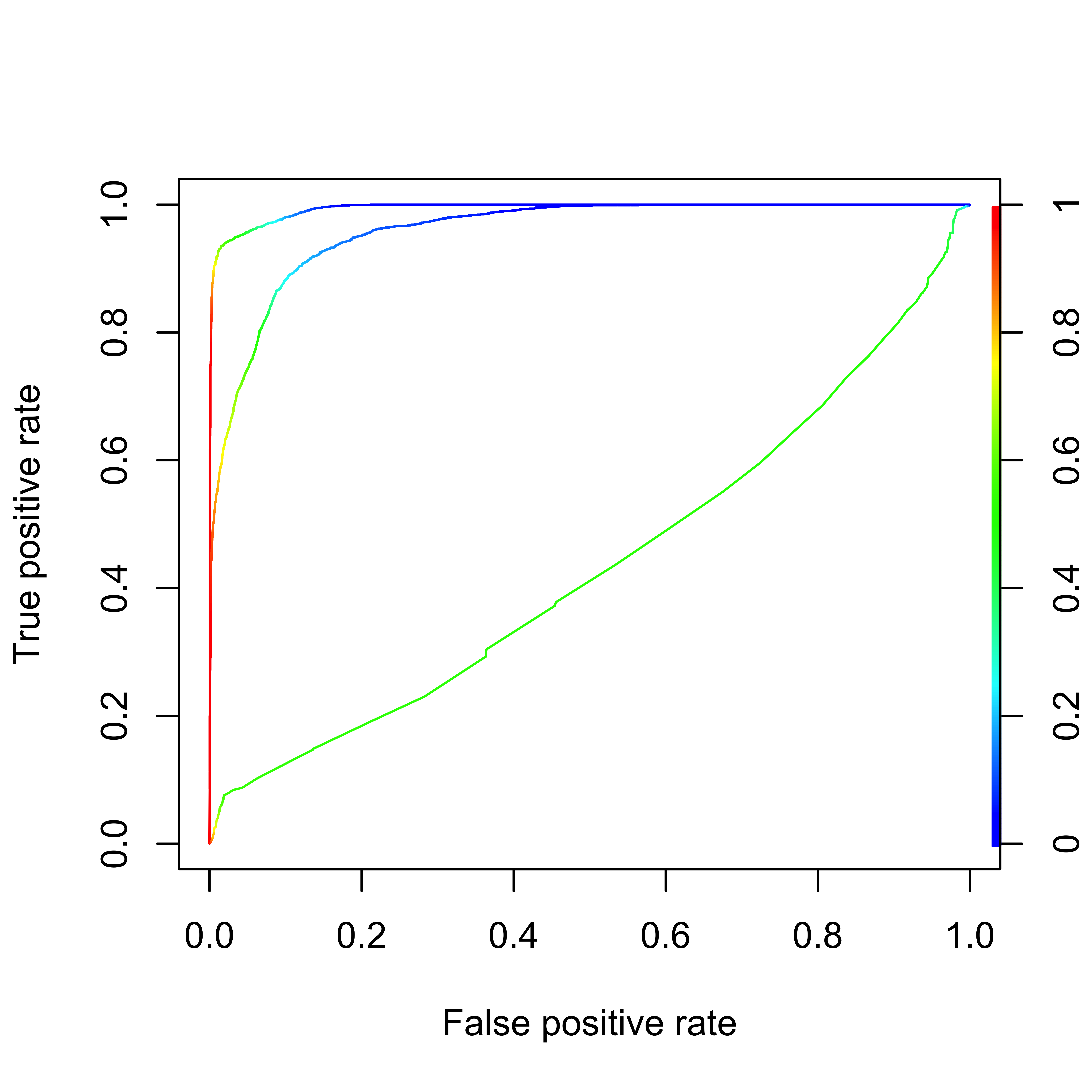


(b) Burden Model.


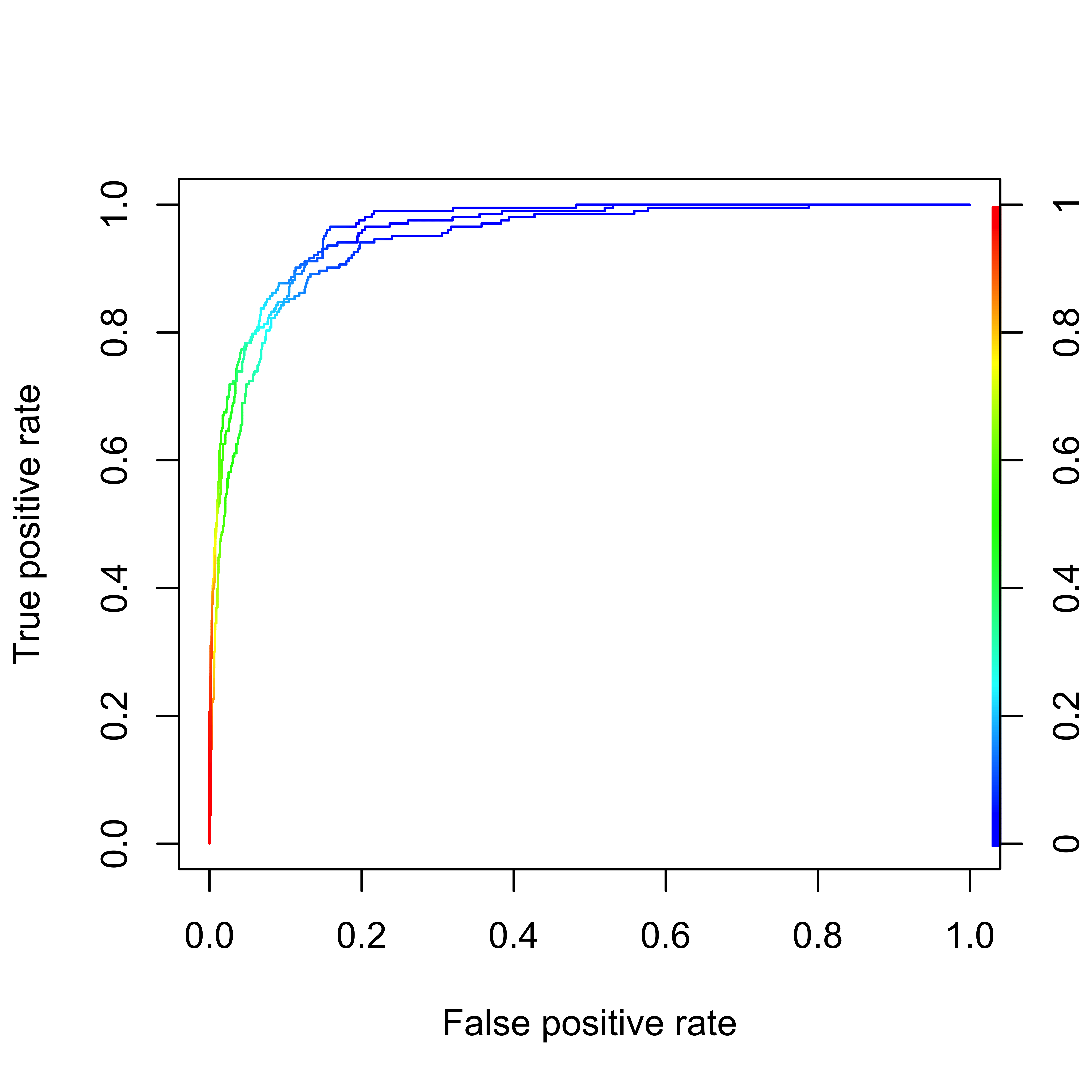


(c) Biologic Syndrome Model.

Note: ROC = Receiver operating characteristic. The color represents specificity (one minus false positive rate). The lines from the top representing the ROC curves of all input variables, own input variables and bias variables to approximate frailty status
