# Supplementary material for: Index or illusion: The case of frailty indices in the Health and Retirement Study
Source: PLoS One. 2018 Jul 18;13(7):e0197859. doi: 10.1371/journal.pone.0197859 (PMC6051600; doi:10.1371/journal.pone.0197859)
Supplement: S4 Appendix — (DOCX) [file pone.0197859.s004.docx]

Appendix 4. Survival curves for the Health and Retirement Study interviewees.


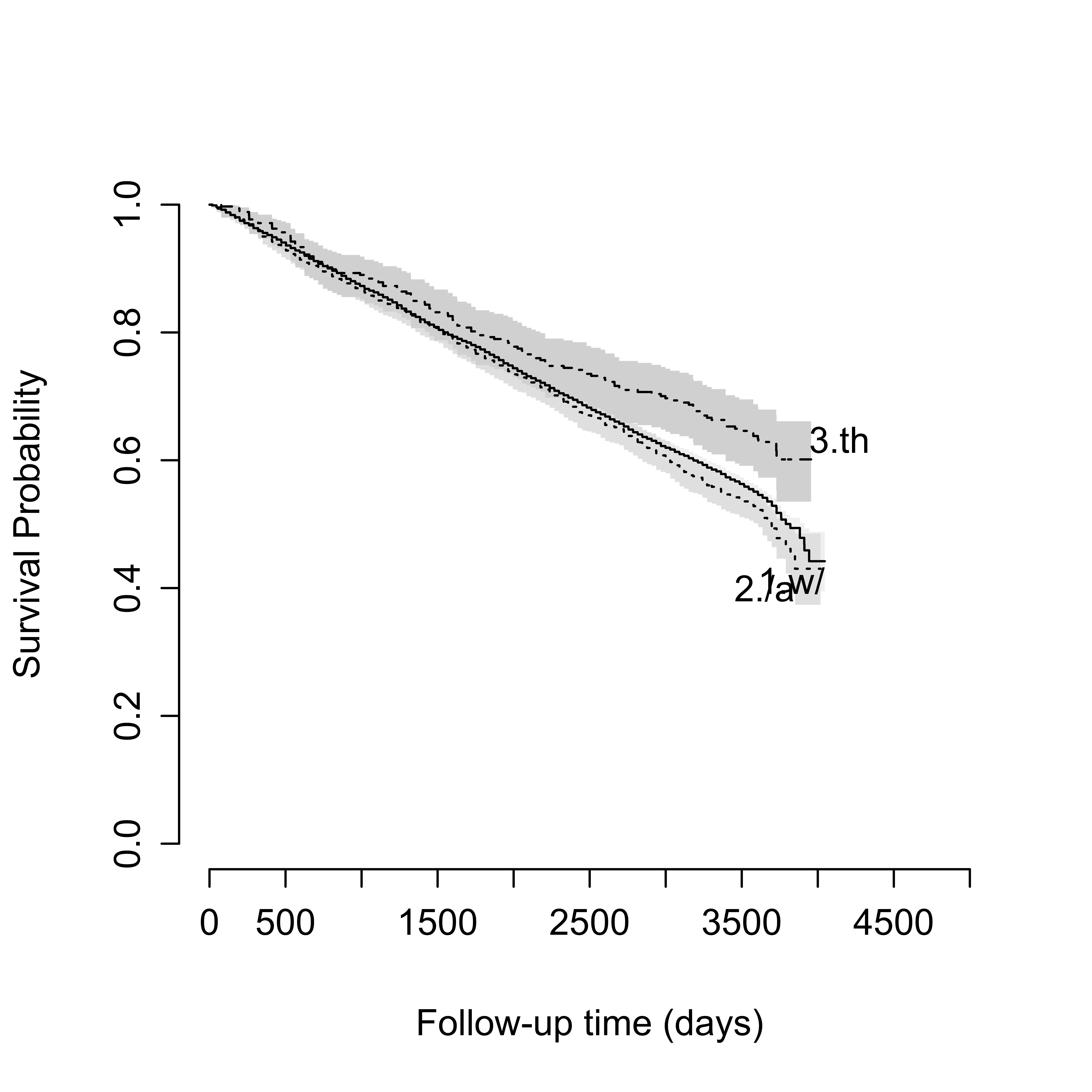


1. Survival curves by race.

Note. 1 = white (solid line), 2 = black/African American (dash line), and 3 = other. Gray area = 95% confidence intervals.


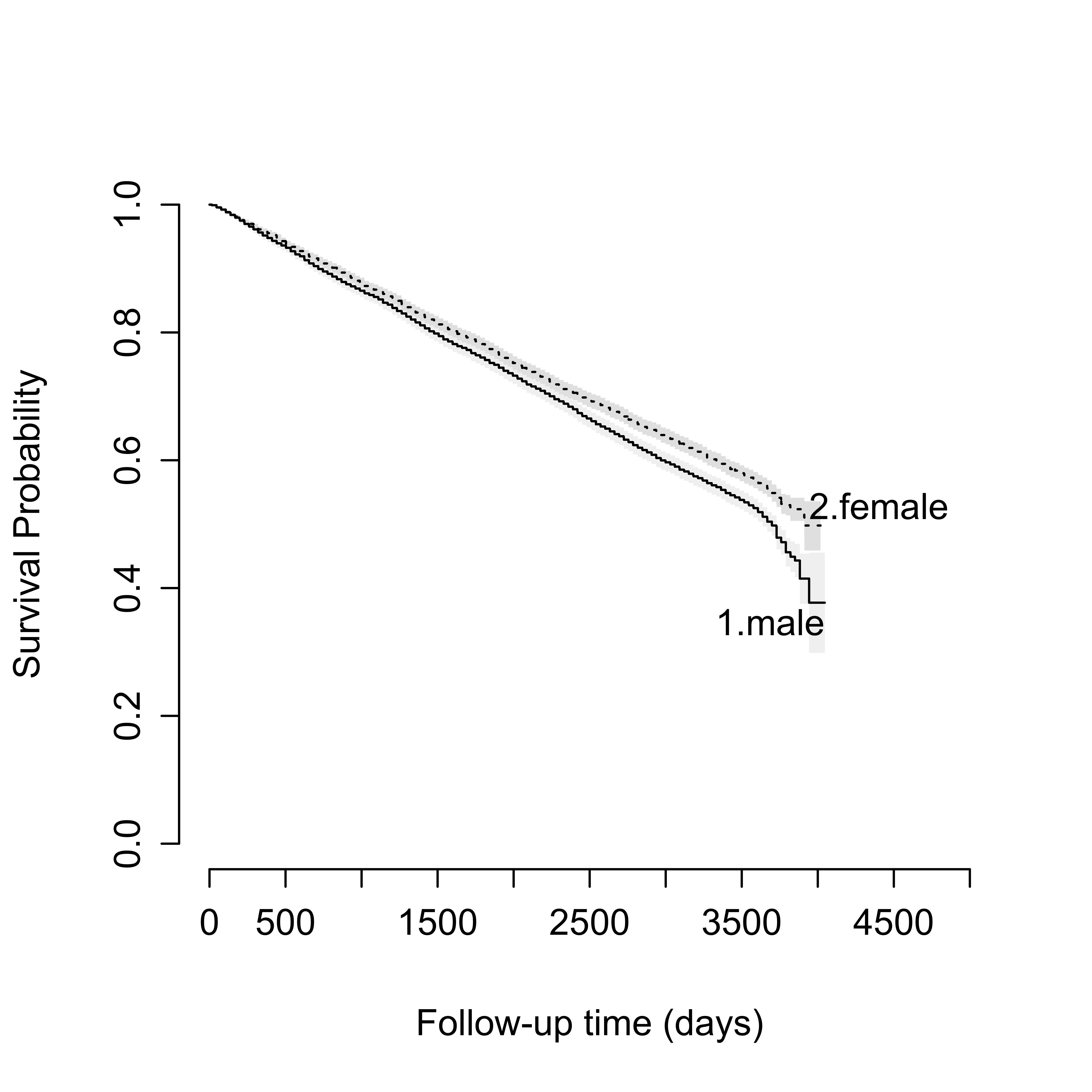


1. Survival curves by sex.

Note. Gray area = 95% confidence intervals.
