# Supplementary material for: Index or illusion: The case of frailty indices in the Health and Retirement Study
Source: PLoS One. 2018 Jul 18;13(7):e0197859. doi: 10.1371/journal.pone.0197859 (PMC6051600; doi:10.1371/journal.pone.0197859)
Supplement: S5 Appendix — (DOCX) [file pone.0197859.s005.docx]

Appendix 5. ROC curves derived from approximating the three frailty statuses with input or bias variables.


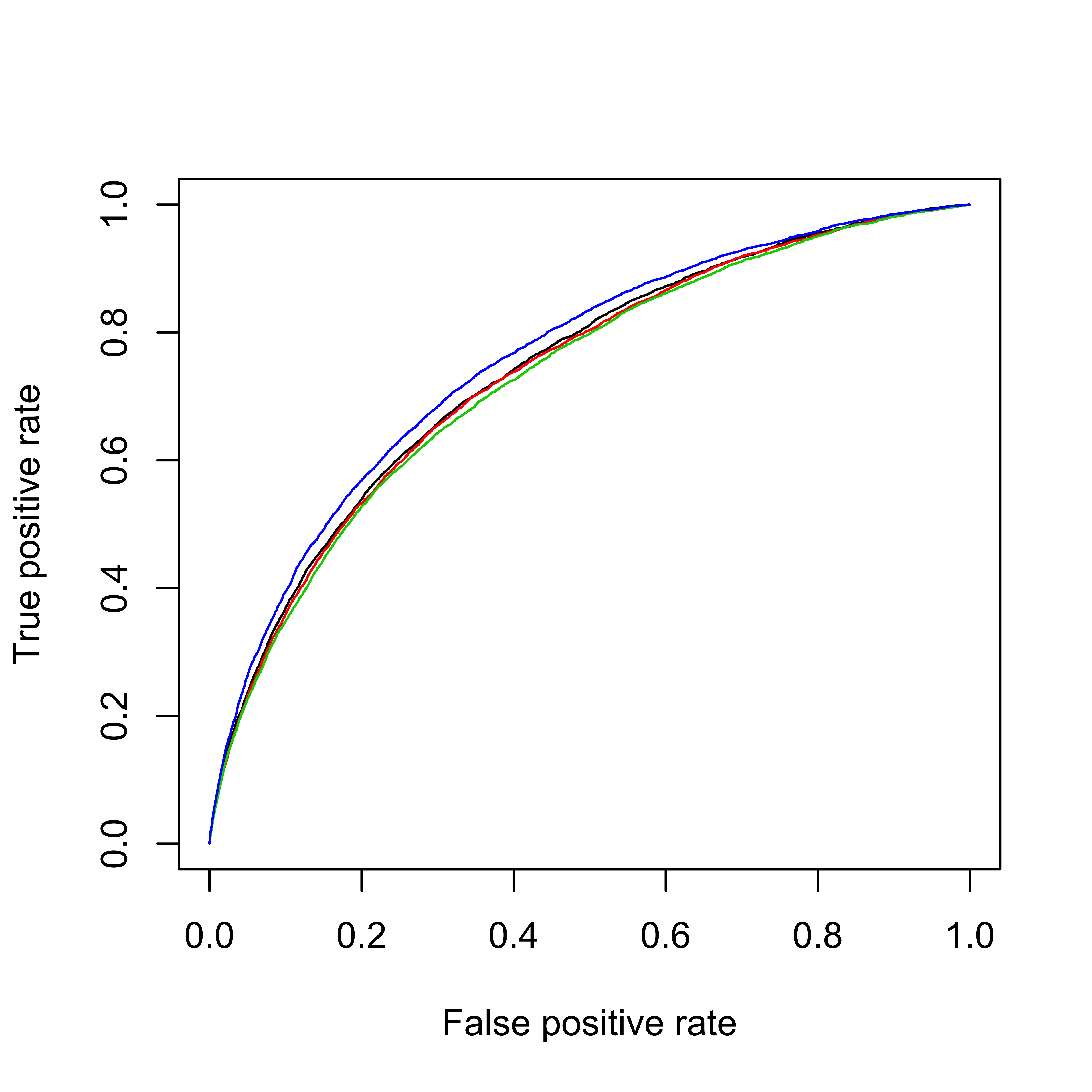


(a) Functional Domains Model.


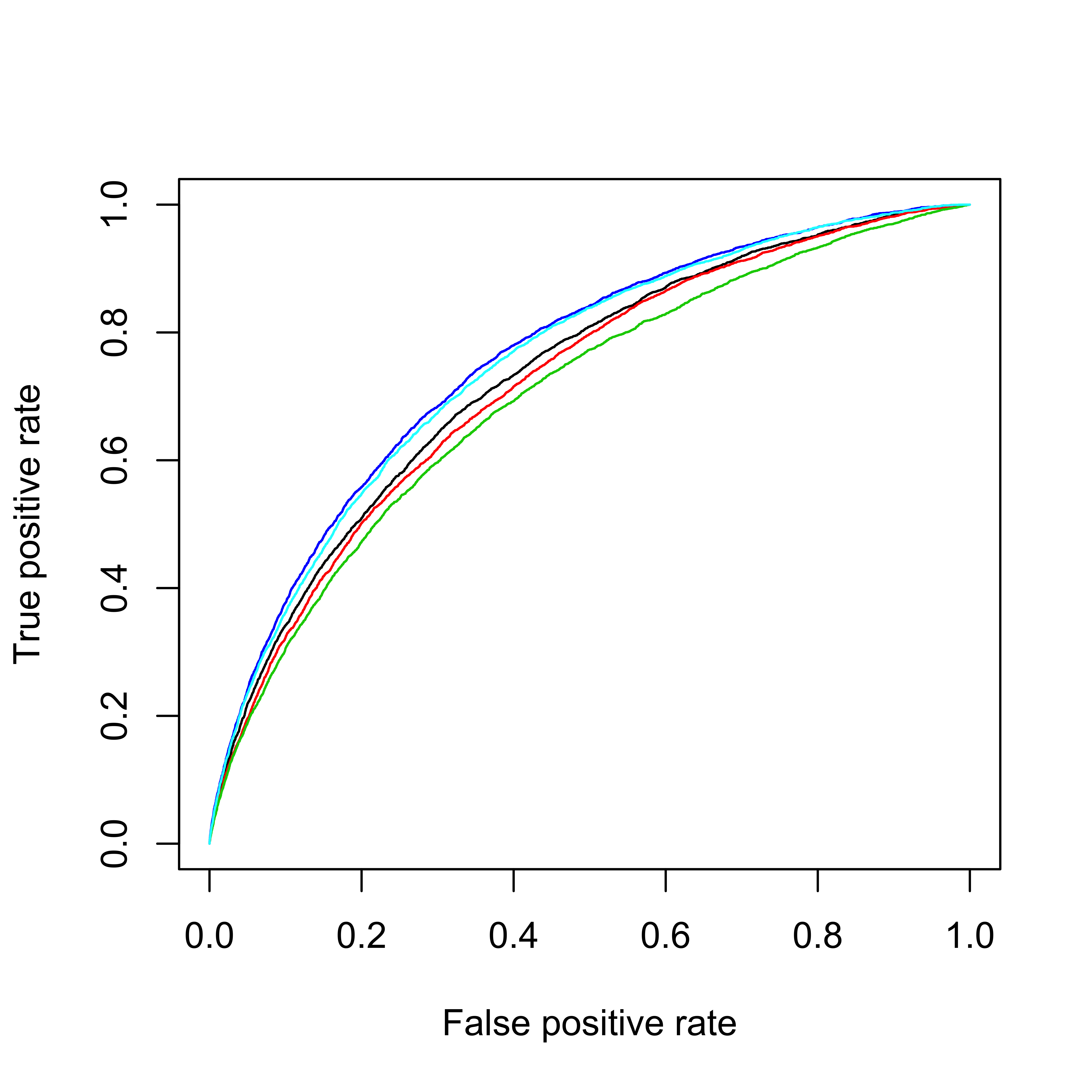


(b) Burden model


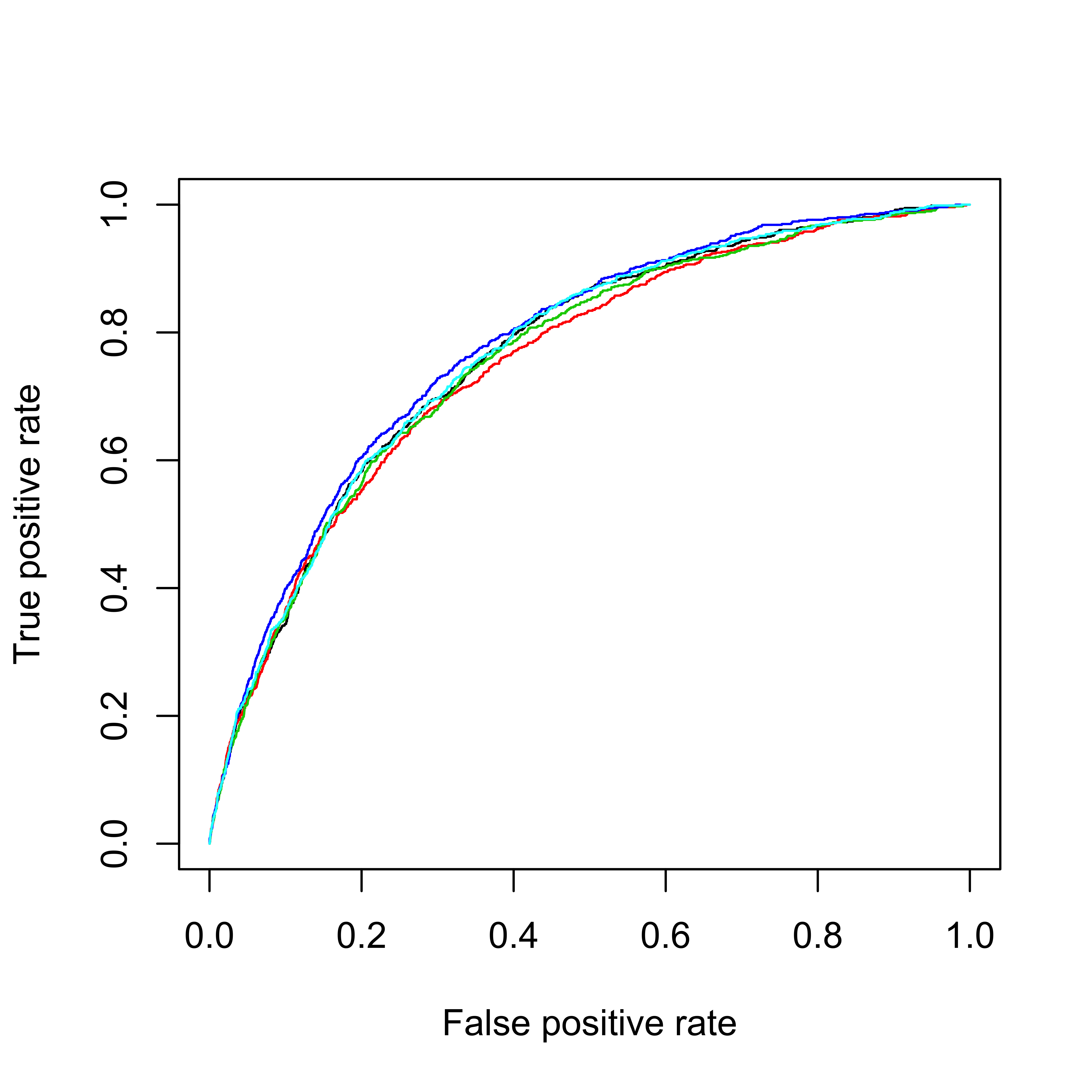


(c) Biological Syndrome model

Note: ROC = Receiver operating characteristic. the upper lines of Functional Domains Model and Burden model representing the logistic models using input variables only; the lower lines of Functional Domains Model and Burden model representing the logistic models using bias variables only. The contrary is true for Biological Syndrome model. See Table 1 for the area-under-curve (AUC) proportions.
