# Supplementary material for: Index or illusion: The case of frailty indices in the Health and Retirement Study
Source: PLoS One. 2018 Jul 18;13(7):e0197859. doi: 10.1371/journal.pone.0197859 (PMC6051600; doi:10.1371/journal.pone.0197859)
Supplement: S6 Appendix — (DOCX) [file pone.0197859.s006.docx]

## Appendix 6. A draft guide to mine index and report mining process

The unexpected magnitude of bias and ubiquitous presence of bias and assumptions motivate us to propose a draft guide to mine innovative indices and report the mining process (Figure 3). First, two of the fundamental assumptions of index need to be well recognized[1]. The weights assigned explicitly declare the relationships between input variables. If the index used for outcome prediction, the relationship designated by weights will remain fixed regardless of the outcome. The actual coefficients of the input variables will change in the same scale simultaneously depending on the outcomes. If the index assumptions are acceptable for the subject matter, the authors are encouraged to continue. Second, we find the lack of disclosure of the assumptions of the three frailty indices central to our findings. There should be clear declaration about the assumptions of the desired index, especially variable inclusion and exclusion criteria, justification of assumptions related to the target population, and how the weights and variables are justified.

Third, the variables need to be processed based on evidence. Categorization of continuous variables and top censoring are not recommended. Once data are processed, there are two potential products that need to be reported and evaluated in the next steps: derived and bias variables. Fourth, the innovative index needs to be evaluated for the relationship with the conceptual framework that motivates the production of the new index. One of the methods is linear approximation to understand how much of the index variance could not be explained by input or derived variables. Fifth, the index created may be used to represent other existing concepts or measurements. It is important to know what the index represent or relate. If there is gold standard, it is easier to properly assess the adequacy and performance of the index. Sixth, the sensitivity and specificity need to be investigated to understand whether the index is able to correctly classify the subjects. Seventh, if the classification error tolerable and the index concept well defined, the index can be considered for other use, especially outcome prediction. We strongly recommend the predictive power of the index compared to those of the input and bias variables. With thorough assessment of using the index as predictor, the authors may suggest methods to use the index for outcome prediction.

## Suggestions to data analysis

There are two suggestions for data analysis. First, data transformation without evidence should be avoided. Dichotomizing continuous variables introduce noise[2] and lead to attrition in predictive power as the three frailty indices show. Data transformation that introduces bias or noise to frailty indices, especially categorization of continuous variables and creation of missing value categorization for three frailty indices, are prevalent and common to epidemiological studies[3]. Even for well-tested indices, such as BMI, it seems preferable to first try continuous scales to predict outcomes and not to first and only use categorized indices for analysis. Categories derived from continuous indices remain useful in the reporting descriptive statistics by groups. Second, data processing should be evidence-based. For example, the conversion between Likert scales and continuous values requires extensive research[4]. Data censoring by setting cut-off values for the sum of input variables needs to be explicitly examined for the loss of interpretability and predictive power.

Figure. Proposed guide to mine innovative index and report the process of index mining.

# References

1. Chao, Y.-S. and C.-J. Wu, *Principal component-based weighted indices and a framework to evaluate indices: Results from the Medical Expenditure Panel Survey 1996 to 2011.* PLoS ONE, 2017. **12**(9): p. e0183997.

2. Barnwell-Menard, J.L., Q. Li, and A.A. Cohen, *Effects of categorization method, regression type, and variable distribution on the inflation of Type-I error rate when categorizing a confounding variable.* Stat Med, 2015. **34**(6): p. 936-49.

3. Rothman, K.J., S. Greenland, and T.L. Lash, *Modern Epidemiology*. 2008, New York, NY: Wolters Kluwer Health/Lippincott Williams & Wilkins.

4. Hasson, D. and B.B. Arnetz, *Validation and Findings Comparing VAS vs. Likert Scales for Psychosocial Measurements.* International Electronic Journal of Health Education, 2005(8): p. 178-192.
